# Year 1 of Medicare’s Accountable Care Organization Realizing Equity, Access, and Community Health Model

**DOI:** 10.1001/jamahealthforum.2025.0724

**Published:** 2025-04-25

**Authors:** Gmerice Hammond, Sunny Lin, Sukruth A. Shashikumar, R. J. Waken, Fengxian Wang, Khavya Avula, Vi-Anh Hoang, Kenton J. Johnston, Karen Joynt Maddox

**Affiliations:** 1 Cardiovascular Division, Department of Medicine, Washington University School of Medicine in St Louis, St Louis, Missouri; 2 Center for Advancing Health Services, Policy and Economics Research, Washington University Institute for Public Health, St Louis, Missouri; 3 Division of General Medical Sciences, Washington University School of Medicine, St Louis, Missouri; 4 Department of Medicine, Brigham and Women’s Hospital, Boston, Massachusetts; 5 Division of Biostatistics, Washington University School of Medicine, St Louis, Missouri

## Abstract

**Importance:**

The US Centers for Medicare & Medicaid Services launched the Accountable Care Organization (ACO) Realizing Equity, Access, and Community Health (REACH) payment model in January 2023. In contrast to prior ACO initiatives, such as the Medicare Shared Savings Program (MSSP), ACO REACH includes equity-focused measures and payment adjustments, including an equity plan and financial risk adjustment for ACOs with higher proportions of underserved beneficiaries. However, it is unknown whether these changes have incented participation from organizations that serve beneficiaries from marginalized communities.

**Objective:**

To compare characteristics between participants in ACO REACH with those in MSSP and the broader pool of Medicare beneficiaries, organizations, and clinicians.

**Design, Setting, and Participants:**

This cross-sectional study included all Medicare beneficiaries clinicians, and ACOs enrolled in fee-for-service Medicare, MSSP, and ACO REACH from January 2022 to January 2023.

**Exposure:**

Enrollment in fee-for-service Medicare, MSSP, or ACO REACH.

**Main Outcomes and Measures:**

Beneficiary, clinician, and ACO characteristics.

**Results:**

In 2023, among 35 801 118 beneficiaries in the overall fee-for-service Medicare program, 18 911 213 (52.8%) were female, and 163 706 (0.5%) were American Indian or Alaska Native, 1 251 553 (3.5%) were Asian or Pacific Islander, 2 952 244 (8.2%) were Black, 2 396 771 (6.7%) were Hispanic, 27 642 765 (77.2%) were White, and 1 394 079 (3.9%) were another race (includes individuals who did not identify with a listed race, including those who self-identified as multiracial) or unknown race. A total of 1 958 881 beneficiaries were attributed to ACO REACH, and 11 340 987 were attributed to MSSP. A total of 132 ACOs participated in ACO REACH, while 456 ACOs participated in the MSSP. Compared with Medicare beneficiaries overall, REACH beneficiaries were older (85 years or older: 14.2% vs 10.3%; standardized mean difference [SMD], 0.44) and more often White (80.2% vs 77.2%) and less often Black (5.9% vs 8.2%) or Hispanic (5.8% vs 6.7%) (SMD, 0.24). REACH beneficiaries were slightly less likely to have Medicare entitlement due to disability (15.2% vs 17.6%) or be dually enrolled (15.1% vs 15.8%) (SMD, 0.07). REACH beneficiaries were less likely to be rural (3.9% vs 8.4%; SMD, 0.19) and less likely to reside in highly vulnerable geographic areas based on the Social Vulnerability Index (27.7% vs 29.4%; SMD, 0.08) compared with beneficiaries overall.

**Conclusions and Relevance:**

These findings suggest that, in its first year, ACO REACH did not achieve its goal of enrolling organizations that serve beneficiaries with high levels of social risk. Without broader participation, ACO REACH is unlikely to achieve its goal of reducing health inequities.

## Key Points

**Question:**

Did the Accountable Care Organization (ACO) Realizing Equity, Access, and Community Health (REACH) initiative enroll a beneficiary population that had high levels of social risk?

**Findings:**

In this cross-sectional analysis including 35.8 million fee-for-service Medicare beneficiaries, along multiple dimensions of social risk, REACH beneficiaries were at significantly lower risk than the overall pool of Medicare beneficiaries, and REACH ACOs were located in less socially vulnerable areas.

**Meaning:**

The ACO REACH initiative did not enroll a high-risk beneficiary population in terms of social risk, which may limit its effectiveness in reducing health inequities in Medicare.

## Teaser

This cross-sectional study compares characteristics between participants in Accountable Care Organization Realizing Equity, Access, and Community Health with Medicare Shared Savings Program and the broader pool of Medicare beneficiaries, organizations, and clinicians.

## Introduction

There are systemic and structural barriers to equitable health care access, quality, and outcomes for individuals from populations made vulnerable to social risk due to multiple factors, including race, ethnicity, socioeconomic status, and location, and the inequities these barriers engender have been widening over time.^1,2,3,4,5,6,7,8,9,10^ American Indian and Alaska Native, Black, and Hispanic individuals, people with low incomes or living in areas with high levels of social vulnerability, and people living in rural areas have a high burden of chronic disease, high rates of hospitalization and emergency department use, and high mortality.^1,2,3,4,5,6,7,8,9,10,11,12,13,14,15^ There is an urgent need to identify strategies to reduce these inequities.

One way that the US Centers for Medicare & Medicaid Services (CMS) has attempted to do so is through novel alternative payment models. Specifically, as a centerpiece for a renewed focus on health equity, CMS introduced the Accountable Care Organization (ACO) Realizing Equity, Access, and Community Health (REACH) initiative, which launched in January 2023.^16^ The ACO REACH program builds on a long history of accountable care under Medicare; the concept of ACOs was first launched at the federal level in 2012. A schematic of the broader Medicare payment landscape, and how REACH fits in, is provided in eFigure 1 in Supplement 1. However, in a departure from those prior CMS payment models,^17,18,19^ to our knowledge, REACH is the first Medicare program to explicitly focus on and prioritize equity. Prior value-based and alternative payment models without an explicit equity focus have not been shown to improve equity for marginalized patients or their clinicians in the Medicare program.^20,21,22,23,24^

In contrast to prior ACO programs, ACO REACH directly aims to advance health equity to bring the benefits of ACOs to underserved communities. Medicare’s largest and longest running ACO program, the Medicare Shared Savings Program (MSSP), has had limited participation among underserved communities, and this has been a limitation of this program’s ability to impact inequities.^24,25,26^ ACO REACH aims to expand participation by making changes intended to attract clinicians, practices, and health systems that care for a high proportion of patients with high social risk. Most importantly, REACH recognizes that achieving good health outcomes among socially higher-risk populations may cost more and adjusts the performance year benchmark upward for ACOs serving higher proportions of beneficiaries who live in highly disadvantaged areas. Other innovations aimed at improving equity include requiring ACOs to collect data on health-related social needs and requiring ACOs to create a health equity plan around a high-priority population.^27^ However, whether the changes made will be sufficient to attract a broader range of clinicians, practices, and health systems as participants than prior ACO programs, including those who care for a higher proportion of high socially and medically at-risk beneficiaries, and, most importantly, whether this will result in tangible reduction in racial or ethnic and other inequities is unknown. While it may take years to assess the program’s impact on outcomes, its success in achieving broader access is crucial to assess in the near term. If access to ACOs is improved under ACO REACH for patients made vulnerable to social risk, then studying its implementation could generate important lessons for broader efforts to address inequities. On the other hand, if ACO REACH fails to improve or even worsens access, it is crucial to identify this early so that policymakers can change the program to meet its intended objectives.

The central aim of this study was therefore to examine the impact of the ACO REACH program on access to ACOs for Medicare beneficiaries by race and ethnicity, rural residence, poverty, or social vulnerability. To do so, we compared participants in ACO REACH with those in Medicare’s largest and most established ACO program, the MSSP, and with the population of Medicare beneficiaries and clinicians overall.

## Methods

### Data and Study Population

We identified ACOs participating in REACH and MSSP using participation files provided by Medicare on the Chronic Condition Data Warehouse. To identify beneficiaries attributed to REACH and MSSP, we used beneficiary attribution files. It is important to note that although beneficiaries are attributed to MSSP ACOs retrospectively at year end based on the tax identification number (TIN) with the plurality of their primary care services, they are attributed to REACH at the beginning of the year based on utilization in the prior year or based on voluntary alignment at any time. Because of these differences in attribution techniques, we compared January 2022 MSSP beneficiaries (retrospectively attributed in 2023 based on visits in 2022) with January 2023 REACH beneficiaries (prospectively attributed in 2023 based on visits in 2022). We linked the attribution files with the Medicare enrollment database to obtain patient characteristics, including county of residence, age, sex, and race and ethnicity. To capture race and ethnicity, we used the Research Triangle Institute race code, which augments race and ethnicity data collected at the time of Social Security enrollment with an algorithm that considers surname and geographic location to increase sensitivity for people who identify as American Indian or Alaska Native, Asian, Black, and Hispanic.^28,29^ We also used these enrollment data to classify each beneficiary’s original reason for entitlement, the presence of dual enrollment in Medicaid, and rurality based on county core-based statistical area coding using county of residence. We excluded beneficiaries missing any of the aforementioned information. We used the county Social Vulnerability Index (SVI) to examine local socioeconomic vulnerability. We used the Chronic Conditions Warehouse algorithms to identify comorbidities.

This study was approved by the Office of Human Research Protection at the Washington University School of Medicine and followed the Strengthening the Reporting of Observational Studies in Epidemiology (STROBE) reporting guideline. The requirement for informed consent was waived due to the deidentified nature of the data.

To characterize clinicians and organizations participating in ACOs, we identified individual clinician and organization national provider identifiers (NPIs) in ACO REACH provider attribution files; for MSSP, we identified NPIs within TINs, where every individual TIN was considered to be a practice. We linked the NPIs with the National Plan and Provider Enumeration System to obtain public information on clinician age, gender, specialty, and location. Using primary taxonomy codes, NPIs were grouped into categories based on a review of the literature of common or expected categories of participants in ACO programs. NPIs were grouped into physician categories (ie, MD and DO), nonphysician categories (eg, MSW, RN), and organizational categories. Physician categories included primary care clinicians (internal medicine, family medicine, and obstetrics and gynecology), hospitalists, mental health, surgery, geriatrics, emergency medicine, hospice, medical students, and all others. Nonphysician NPI categories included advanced practice clinicians, community health workers, physical therapists/occupational therapists, case managers, optometrists, dieticians, dental professionals, chiropractors, nurses/medical assistance/technicians, mental health professionals, pharmacists, and all others. Organizational NPI categories included hospitals, clinics, residential or adult day cares, laboratories, managed care/preferred provider organizations, pharmacy and nonpharmacy dispensing, durable medical equipment, home health organizations, hospice care, end-stage kidney disease treatment, federal or critical access clinics, rural clinics, nursing care agencies, critical access hospitals, and all others. A full list of NPI taxonomies and their categories are included in eTable 3 in Supplement 1.

### Statistical Analysis

We first compared beneficiary characteristics using a Pearson χ^2^ test. Then, we compared REACH and MSSP ACOs on key characteristics, including number of participants, number of unique states and zip codes covered by each ACO, risk sharing, and benefit enhancements. Of note, we could not complete formal statistical testing on some of the organizational elements since the categories are not mutually exclusive and many of the features (benefit enhancements, for example) are dissimilar between the programs. We chose to calculate the number of unique states and zip codes covered by 95% of ACO participants, instead of 100% of participants, to obtain a more realistic characterization of how concentrated participants are. This approach allowed us to drop participants whose official locations were not colocated with other participants due to outdated addresses or second offices in noncontinental US locations. We then created a histogram of each and calculated the median. We plotted the distribution of the SVI in 5% increments using a population pyramid graph approach. Analyses were performed using SAS version 9.4 (SAS Institute) and R Studio version 4.4.1 (Posit PBC).

## Results

### Characteristics of Beneficiaries

Among 35 801 118 beneficiaries in the overall fee-for-service Medicare program in 2023, among 35 801 118 beneficiaries in the overall fee-for-service Medicare program, 18 911 213 (52.8%) were female, and 163 706 (0.5%) were American Indian or Alaska Native, 1 251 553 (3.5%) were Asian or Pacific Islander, 2 952 244 (8.2%) were Black, 2 396 771 (6.7%) were Hispanic, 27 642 765 (77.2%) were White, and 1 394 079 (3.9%) were another race (includes individuals who did not identify with a listed race, including those who self-identified as multiracial) or unknown race. A total 1 958 881 beneficiaries were attributed to 132 ACO REACH ACOs, and 11 340 987 were attributed to 456 MSSP ACOs (Table 1).

**Table 1.** Beneficiary Characteristics for the Realizing Equity, Access, and Community Health (REACH) and Medicare Shared Savings Program (MSSP) Accountable Care Organizations and All Fee-for-Service Medicare Beneficiaries

Characteristic	No. (%)			SMD	
	REACH	MSSP	All fee-for-service	REACH vs MSSP	REACH vs fee-for-service Medicare
Total beneficiaries, No.	1 990 183	10 558 446	35 801 118	NA	NA
Age, y					
<65	150 889 (7.6)	851 481 (8.1)	3 861 640 (10.8)	0.02	0.44
65-74	856 571 (43.0)	4 576 499 (43.3)	18 726 664 (52.3)		
75-84	701 069 (35.2)	3 694 473 (35.0)	9 530 224 (26.6)		
≥85	281 654 (14.2)	1 435 993 (13.6)	3 682 590 (10.3)		
Sex					
Male	855 021 (43.0)	4 555 607 (43.2)	16 889 905 (47.2)	<0.01	0.09
Female	1 135 162 (57.0)	6 002 839 (56.8)	18 911 213 (52.8)		
Race and ethnicity					
American Indian or Alaska Native	3599 (0.2)	18 423 (0.2)	163 706 (0.5)	0.37	0.24
Asian or Pacific Islander	91 977 (4.6)	241 373 (2.3)	1 251 553 (3.5)		
Black	117 445 (5.9)	64 5017 (6.1)	2 952 244 (8.2)		
Hispanic	114 832 (5.8)	411 448 (3.9)	2 396 771 (6.7)		
Non-Hispanic White	1 596 572 (80.2)	8 907 565 (84.4)	27 642 765 (77.2)		
Other race^a^	17 908 (0.9)	79 538 (0.8)	347 763 (1.0)		
Unknown race	47 850 (2.4)	255 082 (2.4)	1 046 316 (2.9)		
Reason for Medicare entitlement					
Disability	302 105 (15.2)	1 687 631 (16.0)	6 284 282 (17.6)	0.02	0.07
ESKD	4396 (0.2)	26 781 (0.2)	151 415 (0.4)		
Both disability and ESKD	2064 (0.1)	11 836 (0.1)	44 253 (0.1)		
Dual eligible	300 492 (15.1)	1 332 521 (12.6)	5 654 015 (15.8)	0.07	0.02
Rural	77 887 (3.9)	893 589 (8.5)	3 002 374 (8.4)	0.20	0.19
SVI quartile					
First (lowest SVI)	286 521 (14.4)	1 836 818 (17.4)	5 583 303 (15.6)	0.09	0.08
Second quartile	579 936 (29.1)	3 012 165 (28.5)	9 426 017 (26.3)		
Third quartile	571 556 (28.7)	3 042 247 (28.8)	10 279 421 (28.7)		
Fourth (highest SVI)	552 170 (27.7)	2 667 216 (25.3)	10 512 377 (29.4)		
Any chronic condition	1 963 311 (98.6)	10 465 157 (99.1)	26 002 167 (72.6)	0.05	0.80

Abbreviations: ESKD, end-stage kidney disease; NA, not applicable; SMD, standardized mean difference; SVI, Social Vulnerability Index.

^a^ Includes individuals who did not identify with a listed race, including those who self-identified as multiracial.

**Figure 1.**
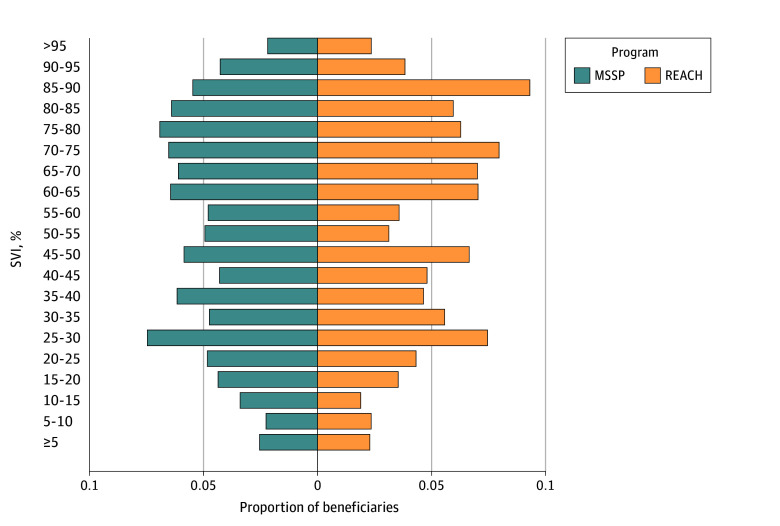
Social Vulnerability Index (SVI) Population Density Tree for the Realizing Equity, Access, and Community Health (REACH) and Medicare Shared Savings Program (MSSP) Accountable Care Organizations

Compared with Medicare beneficiaries overall, REACH beneficiaries were at lower risk in terms of most sociodemographic characteristics. REACH beneficiaries were older (85 years or older: 14.2% vs 10.3%; standardized mean difference [SMD], 0.44) and were more often White (80.2% vs 77.2%) and less often Black (5.9% vs 8.2%) or Hispanic (5.8% vs 6.7%) (SMD, 0.24). REACH beneficiaries were slightly less likely to have Medicare entitlement due to disability (15.2% vs 17.6%) and were slightly less frequently dually enrolled (15.1% vs 15.8%) (SMD, 0.07). REACH beneficiaries were markedly less likely to live in a rural area (3.9% vs 8.4%; SMD, 0.19) and slightly less likely to reside in highly vulnerable geographic areas based on the SVI (27.7% vs 29.4%; SMD, 0.08) compared with Medicare beneficiaries overall (Table 1; Figure 1). REACH beneficiaries were more likely to have all 10 of the chronic conditions we examined than the overall fee-for-service population (eTable 2 in Supplement 1).

Comparing REACH with MSSP beneficiaries, there were generally smaller differences in terms of age (eg, the proportion of beneficiaries older than 85 years was 14.2% for REACH and 13.6% for MSSP), race and ethnicity (eg, the proportion of beneficiaries who identified as non-Hispanic White was 80.2% for REACH and 84.4% for MSSP), eligibility due to disability (15.2% for REACH vs 16.0% for MSSP), and dual enrollment (15.1% for REACH vs 12.6% for MSSP), with REACH beneficiaries at slightly higher risk than MSSP beneficiaries in terms of the sociodemographic risk indicators (Table 1). Medical comorbidities were similar between the 2 groups (eTable 2 in Supplement 1).

### Characteristics of REACH ACOs

REACH ACOs tended to be smaller than MSSP ACOs; the mean (SD) volume of attributed beneficiaries was 14 840 (17 861) for REACH ACOs compared with 24 870 (29 910) for MSSP ACOs (Table 2). In terms of clinicians, 6% of REACH ACOs had 50 or fewer participating clinicians and 8% had 51 to 100 clinicians compared with 1% and 4%, respectively, for MSSP ACOs. Similarly, REACH ACOs had a smaller geographic footprint, with a median (range) representation of 95% of its NPIs within 231 (9-2211) unique zip codes and 18 (1-50) unique states compared with 674 (13-4302) and 32 (1-50), respectively, for MSSP ACOs (eFigure 2 in Supplement 1). A total of 82% of REACH ACOs selected the higher-risk risk-sharing option compared with 67% of MSSP ACOs, and a higher proportion of REACH ACOs elected the 3-day skilled nursing facility rule waiver (79% vs 35%).

**Table 2.** Characteristics of the Realizing Equity, Access, and Community Health (REACH) and Medicare Shared Savings Program (MSSP) Accountable Care Organizations (ACOs)

Characteristic	No. (%)	
	REACH (n = 132)	MSSP (n = 456)
Mean beneficiaries per ACO, No.	14 840	24 870
ACO size, No. of participants		
≤50	8 (6)	5 (1)
51-100	11 (8)	17 (4)
101-500	49 (37)	127 (28)
501-1000	23 (17)	90 (20)
≥1001	41 (31)	217 (48)
Geographic spread accounting for 95% of participants, median (range)		
Unique states	18 (1-50)	32 (1-50)
Unique zip codes	231 (9-2211)	674 (13-4302)
Risk-sharing option		
Lower risk	24 (18)	151 (33)
Higher risk	108 (82)	305 (67)
Benefit enhancement of 3-d SNF rule waiver	104 (79)	160 (35)
Previously enrolled in GPDC model	84 (64)	NA

Abbreviations: GPDC, Global and Professional Direct Contracting; NA, not applicable; SNF, skilled nursing facility.

**Figure 2.**
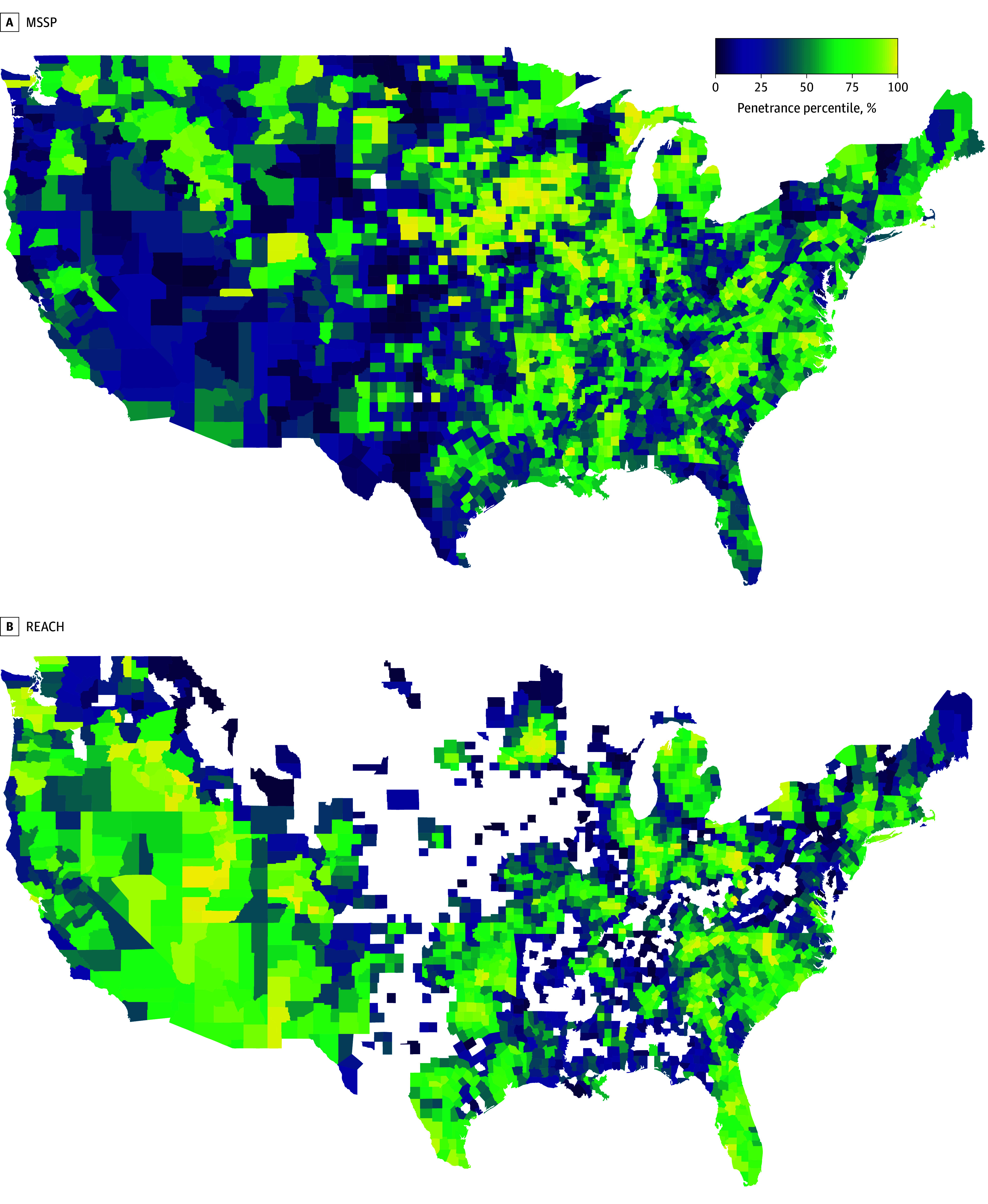
Geographic Presence of the Realizing Equity, Access, and Community Health (REACH) and Medicare Shared Savings Program (MSSP) Accountable Care Organizations

Among characteristics specific to REACH ACOs, 84 REACH ACOs (64%) were retained from the previous Global and Professional Direct Contracting model. A total of 105 (79.6%) were standard ACOs, while 14 (10.6%) were classified as high-needs ACOs (focusing on a specific medically high-risk population) and 13 (9.8%) as new-entrant ACOs (lacking prior ACO experience) (eTable 1 in Supplement 1). The most commonly used capitation mechanism across these ACOs was a combination of primary care capitation and the advance payment option (64 of 132 [48.5%]). Benefit enhancements were used broadly; part B cost-sharing was the most frequently used benefit enhancement, as elected by 101 REACH ACOs (76.5%), and concurrent care for hospice the least frequent, only elected in 46 (34.8%). REACH ACOs were more prevalent in the West compared with MSSP ACOs and less prevalent in the Midwest (Figure 2).

There were 224 410 NPIs listed as affiliated with REACH ACOs, of which 193 119 (86.1%) were clinicians and the remaining 31 291 (13.9%) were organizational. There were 934 131 NPIs listed as affiliated with MSSP ACOs, of which 930 424 (99.6%) were clinicians, 1930 (0.2%) were organizational, and the remaining 1777 (0.2%) were missing a primary taxonomy code (Table 3; eTable 4 in Supplement 1).

**Table 3.** Participant Characteristics for the Realizing Equity, Access, and Community Health (REACH) and Medicare Shared Savings Program (MSSP) Accountable Care Organizations (ACOs) and All National Plan and Provider Enumeration System (NPPES)

Characteristic	No. (%)		
	REACH ACO	MSSP ACO	All NPPES
Type of provider			
Nonorganizational	193 119 (86)	930 424 (>99)	6 710 095 (89)
Organizational	31 291 (14)	1930 (<1)	853 459 (11)
Missing primary taxonomy code	0	1777 (<1)	258 980 (3)
Total	224 410 (100)	934 131 (100)	7 822 534 (100)
Nonorganizational providers			
Physicians (eg, MD, DO)			
Primary care (eg, internal medicine, family medicine, obstetrician/gynecologist)	54 629 (28)	228 743 (25)	569 804 (8)
Mental health	2589 (1)	14 809 (2)	72 605 (1)
Surgery	6154 (3)	45 716 (5)	112 802 (2)
Other	49 051 (26)	304 819 (33)	1 042 293 (16)
Advanced practice clinician (eg, physician assistant, nurse practitioner, clinical nurse specialist)	56 238 (29)	251 798 (27)	616 730 (9)
Physical therapy/occupation therapy	8153 (4)	11 736 (1)	484 715 (7)
Nurse, assistants, aides, and technicians	7002 (4)	42 952 (5)	897 354 (13)
Mental health (eg, medical social workers, counselors)	6458 (3)	17 616 (2)	1 352 586 (20)
Other	2845 (2)	12 235 (1)	1 561 206 (23)
Total	193 119 (100)	930 424 (100)	6 710 095 (100)

Among nonorganizational provider NPIs affiliated with REACH ACOs, 28% were identified as primary care physicians, compared with 25% of MSSP ACOs and only 8% of National Plan and Provider Enumeration System nonorganizational providers. REACH ACOs had a lower proportion of surgical specialists than MSSP ACOs (3% vs 5%) and a higher proportion than the overall clinician pool (2%). REACH ACOs had a lower proportion of emergency care physicians than MSSP ACOs (2% vs 4%) and a higher proportion than the overall clinician pool (1%). REACH ACOs had a similar proportion of advanced practice clinicians compared with MSSP ACOs (29% and 27%, respectively), although both were considerably higher than the overall clinician pool (9%). REACH and MSSP ACOs had lower proportions of clinicians classified as mental health professionals (social workers, clinical psychologists), comprising 3% in REACH ACOs, 2% in MSSP ACOs, and 20% in the overall clinician pool (Table 3).

## Discussion

We evaluated beneficiary, organization, and provider characteristics of ACO REACH and compared beneficiary characteristics between REACH ACOs, MSSP ACOs, and the broader pool of Medicare beneficiaries. We found that REACH beneficiaries were at significantly lower risk than the overall pool of Medicare beneficiaries along multiple dimensions of social risk. These findings suggest that ACO REACH did not, at least in its first year, achieve its goal of enrolling practices that serve beneficiaries with higher levels of social risk.

Despite ACO REACH’s explicit focus on equity, REACH beneficiaries were at lower risk on every dimension of social risk examined compared with the broader pool of Medicare beneficiaries, including Black race or Hispanic ethnicity, disability, dual enrollment, rurality, and SVI. In most ways, REACH beneficiaries looked more similar to MSSP beneficiaries, who have previously been shown to be at lower risk than Medicare beneficiaries at large.^30,31,32^ This raises concerns regarding the program’s ability to effectively enroll and serve populations most vulnerable to social risk factors, thereby limiting its potential impact on health equity. While MSSP beneficiaries also demonstrate lower social risk profiles than the Medicare population at large, the explicit goal of ACO REACH to address health disparities necessitates a more comprehensive approach to enrollment and program implementation. Our findings suggest that up-front investments may be needed for clinician practices to participate in ACO REACH, which CMS has begun rolling out, or other strategies to encourage participation among high-risk groups.^33^

It is also notable that REACH (and MSSP) beneficiaries were at lower risk along every examined line of social risk but were coded as having more chronic conditions than the overall fee-for-service population. One reason could be because attribution to an ACO requires contact with the health care system and thus selects for a group of patients with chronic or acute disease in any given year. REACH beneficiaries were also older, which supports this hypothesis. However, it could also suggest upcoding within REACH and MSSP, which carries financial benefits due to the manner in which ACOs are compensated.^34^ CMS has introduced coding growth caps to try to limit upcoding in both REACH and MSSP, and it will be important to track changes in coded comorbidities as the program matures.^35^

The clinician makeup of REACH is encouraging, to the degree that a high proportion of primary care physicians and advanced practice clinicians signal an investment in team-based care and a focus on care coordination. REACH participants’ requirement to complete equity plans and other equity-focused programmatic elements are intended to incent innovation in care delivery with a focus on improving equity. Whether the combination of REACH incentives and these staffing arrangements will lead to meaningful improvements in health outcomes or reduction in health inequities remains to be seen but is important to monitor closely going forward.

Finally, the capitation arrangements, risk-sharing options, and benefit enhancements elected by REACH participants are notable. In general, the amount of risk assumed is higher in REACH than in MSSP, both in terms of the risk-sharing option and the move toward capitated payments. However, only about one-quarter of REACH ACOs elected the total costs of care version of capitation (which includes capitated payments meant to cover both primary and specialty care), with the remainder electing primary care capitation alone. This may suggest that there may be fewer perceived incentives or opportunities to engage with specialists or facilities to reduce unnecessary utilization more broadly. It may also suggest that clinicians have concerns about inadequate risk adjustment for risk factors more common in beneficiaries with multiple social, behavioral, and health risk factors, a problem that has been well-documented in other alternative payment model programs.^36,37,38,39^ The financial performance of ACOs that elected to receive total vs primary care capitation is an important area for future work.

Lastly, the benefit enhancements in REACH were taken up fairly broadly, with nearly two-thirds electing all but the concurrent care for hospice benefit enhancement. Some of these enhancements are clearly aimed at equity considerations, such as the Part B cost-sharing waiver, which allows ACOs to cover certain cost-sharing elements on beneficiaries’ behalf, a clear benefit for individuals with lower income. Others may impact equity more indirectly. For example, allowing more flexibility in the use of advanced practice clinicians may help beneficiaries who need more social work or other support services. Waiving the 3-day stay requirement for skilled nursing facility services, a waiver that had notably higher uptake among REACH than MSSP ACOs, could reduce inequities by protecting beneficiaries from the financial consequences of uncovered but necessary rehabilitation care after a hospital stay that does not meet the 3-day requirement.^40^ Future qualitative and quantitative work should examine how these waivers facilitate innovative and flexible care delivery to determine if any should be pursued more broadly.

### Limitations

This study has limitations. We only examined 1 year of beneficiary enrollment data, since the ACO REACH program only launched in January 2023, and we did not examine patient-level outcomes. We did not have data on each organization’s equity plan or the high-priority population(s) they selected. Program files available on the Virtual Research Data Center for REACH and MSSP ACOs differ somewhat in the organizational information that is included, because REACH participation is indicated at the NPI level while MSSP participation is indicated at the TIN level. Therefore, comparisons between organizational characteristics could not be tested formally and should be interpreted with that in mind. Furthermore, in this dataset, self-reported race and ethnicity were classified by the Research Triangle Institute race code, which is considered valid for identifying non-Hispanic Black and non-Hispanic White beneficiaries.^28^ However, Research Triangle Institute race coding has been found to be less valid for identifying beneficiaries who are Hispanic of any race, non-Hispanic American Indian and Alaskan Native, non-Hispanic Asian, or Native Hawaiian or Other Pacific Islander due to low sensitivity, possibly leading to potential bias in outcome reporting by race and ethnicity for these groups.^28^

## Conclusions

In this cross-sectional study, ACO REACH beneficiaries had significantly lower social risk profiles than the general fee-for-service population. This underscores the need for targeted interventions to enhance enrollment among patient populations at high risk of health disparities. The lack of broader participation from vulnerable populations could limit the potential impacts of other equity elements of the ACO REACH program in its overarching goal of reducing health inequities. Policy and programmatic modifications are imperative to address enrollment challenges and ensure that ACO REACH effectively serves those most in need.

## Notes

Supplement 1. **eTable 1.** REACH ACO Characteristics **eTable 2.** Beneficiary Chronic Condition Characteristics for REACH, MSSP, and All FFS Medicare **eTable 3.** NPI Types **eTable 4.** Expanded Participant Characteristics for ACO REACH, MSSP, and All NPPES **eFigure 1.** Operational Landscape of Medicare Value-Based Payment Models **eFigure 2.** Histogram of Number of Unique States and Zip Codes in Each ACO Accounting for 95% of All NPIs, REACH, vs MSSP
eReferences.

Supplement 2. Data Sharing Statement
