# Supplementary material for: Year 1 of Medicare’s Accountable Care Organization Realizing Equity, Access, and Community Health Model
Source: JAMA Health Forum. 2025 Apr 25;6(4):e250724. doi: 10.1001/jamahealthforum.2025.0724 (PMC12032566; doi:10.1001/jamahealthforum.2025.0724)
Supplement: Supplement 1. — eTable 1. REACH ACO Characteristics eTable 2. Beneficiary Chronic Condition Characteristics for REACH, MSSP, and All FFS Medicare eTable 3. NPI Types eTable 4. Expanded Participant Characteristics for ACO REACH, MSSP, and All NPPES eFigure 1. Operational Landscape of Medicare Value-Based Payment Models eFigure 2. Histogram of Number of Unique States and Zip Codes in Each ACO Accounting for 95% of All NPIs, REACH, vs MSSP eReferences. [file jamahealthforum-e250724-s001.pdf]

# Supplemental Online Content

Hammond G, Lin S, Shashikumar SA, et al. Year 1 of Medicare's Accountable Care Organization Realizing Equity, Access, and Community Health Model. *JAMA Health Forum*. 2025;6(4):e250724. doi:10.1001/jamahealthforum.2025.0724

**eTable 1.** REACH ACO Characteristics
**eTable 2.** Beneficiary Chronic Condition Characteristics for REACH, MSSP, and All FFS Medicare
**eTable 3.** NPI Types
**eTable 4.** Expanded Participant Characteristics for ACO REACH, MSSP, and All NPPES
**eFigure 1.** Operational Landscape of Medicare Value-Based Payment Models
**eFigure 2.** Histogram of Number of Unique States and Zip Codes in Each ACO Accounting for 95% of All NPIs, REACH, vs MSSP
**eReferences.**

This supplementary material has been provided by the authors to give readers additional information about their work.

© 2025 Hammond G et al. *JAMA Health Forum*.

**eTable 1.** REACH ACO Characteristics

| | **REACH (n=132)** |
|---|---|
| **ACO type** | |
| **Standard ACOs** | 105 (80%) |
| **High-needs ACOs** | 14 (11%) |
| **New entrant ACOs** | 13 (10%) |
| **Capitation mechanism** | |
| **PCC**[a] | 38 (29%) |
| **PCC + APO**[a] | 64 (48%) |
| **TCC**[a] | 30 (23%) |
| **Benefit enhancements** | |
| **Home Health Homebound waiver** | 79 (60%) |
| **Post-discharge home visits** | 84 (64%) |
| **Care management home visits** | 83 (63%) |
| **Telehealth** | 82 (62%) |
| **Concurrent care for hospice** | 46 (35%) |
| **Part B cost sharing support** | 101 (76%) |
| **Chronic disease management reward** | 91 (69%) |
| **Nurse practitioner/physician assistant services** | 79 (60%) |

[a]PCC: Primary Care Capitation, APO: Advanced Payment Option, TCC: Total Care Capitation

© 2025 Hammond G et al. *JAMA Health Forum*.

**eTable 2.** Beneficiary Chronic Condition Characteristics for REACH, MSSP, and All FFS Medicare

| | REACH | MSSP | All FFS | SMD, REACH v MSSP | SMD, REACH v FFS |
|---|---|---|---|---|---|
| **Hypertension** | 1,537,622 (77.3%) | 8,223,713 (77.9%) | 19,708,840 (55.0%) | 0.014 | 0.485 |
| **Hyperlipidemia** | 1,637,801 (82.3%) | 8,645,570 (81.9%) | 20,136,364 (56.2%) | 0.010 | 0.588 |
| **Diabetes** | 646,503 (32.5%) | 3,262,313 (30.9%) | 8,227,561 (23.0%) | 0.034 | 0.575 |
| **Heart failure and non-ischemic heart disease** | 274,519 (13.8%) | 1,436,454 (13.6%) | 3,548,379 (9.9%) | 0.006 | 0.121 |
| **Ischemic heart disease** | 581,265 (29.2%) | 3,073,832 (29.1%) | 7,283,973 (20.4%) | 0.002 | 0.205 |
| **Acute myocardial infarction** | 43,321 (2.2%) | 238,383 (2.3%) | 595,088 (1.7%) | 0.007 | 0.036 |
| **Stroke/TIA** | 175,489 (8.8%) | 920,513 (8.7%) | 2,238,915 (6.2%) | 0.004 | 0.098 |
| **COPD** | 358,368 (18.0%) | 1,892,172 (17.9%) | 4,514,532 (12.6%) | 0.003 | 0.150 |
| **Cancer** | 293,057 (14.7%) | 1,615,838 (15.3%) | 3,674,839 (10.3%) | 0.017 | 0.133 |
| **Alzheimer's** | 181,503 (9.1%) | 828,173 (7.8%) | 2,295,528 (6.4%) | 0.047 | 0.101 |

© 2025 Hammond G et al. *JAMA Health Forum*.

**eTable 3.** NPI Types

| Code | Taxonomy | Type |
|---|---|---|
| 405300000X | Prevention Professional | Ancillary |
| 376J00000X | Homemaker | Ancillary |
| 376G00000X | Nursing Home Administrator | Ancillary |
| 374T00000X | Religious Nonmedical Nursing Personnel | Ancillary |
| 374J00000X | Doula | Ancillary |
| 3747P1801X | Personal Care Attendant | Ancillary |
| 3747A0650X | Attendant Care Provider | Ancillary |
| 251300000X | Local Education Agency (LEA) | Ancillary |
| 246YC3302X | Physician Office Based Coding Specialist | Ancillary |
| 237700000X | Hearing Instrument Specialist | Ancillary |
| 235Z00000X | Speech-Language Pathologist | Ancillary |
| 2279H0200X | Home Health Registered Respiratory Therapist | Ancillary |
| 227900000X | Registered Respiratory Therapist | Ancillary |
| 2278C0205X | Critical Care Certified Respiratory Therapist | Ancillary |
| 225XM0800X | Mental Health Occupational Therapist | Ancillary |
| 225A00000X | Music Therapist | Ancillary |
| 2255A2300X | Athletic Trainer | Ancillary |
| 225000000X | Orthotic Fitter | Ancillary |
| 224Y00000X | Clinical Exercise Physiologist | Ancillary |
| 224P00000X | Prosthetist | Ancillary |
| 224L00000X | Pedorthist | Ancillary |
| 222Q00000X | Developmental Therapist | Ancillary |
| 177F00000X | Lodging Provider | Ancillary |
| 175L00000X | Homeopath | Ancillary |
| 175F00000X | Naturopath | Ancillary |
| 174N00000X | Lactation Consultant (Non-RN) | Ancillary |
| 174H00000X | Health Educator | Ancillary |
| 1744R1103X | Research Study Abstracter/Coder | Ancillary |
| 1744R1102X | Research Study Specialist | Ancillary |
| 173F00000X | Sleep Specialist (PhD) | Ancillary |
| 172P00000X | Naprapath | Ancillary |
| 172A00000X | Driver | Ancillary |
| 171WH0202X | Home Modifications Contractor | Ancillary |
| 171W00000X | Contractor | Ancillary |
| 171R00000X | Interpreter | Ancillary |

© 2025 Hammond G et al. *JAMA Health Forum*.

| | | |
|---|---|---|
| 171100000X | Acupuncturist | Ancillary |
| 170300000X | Genetic Counselor (M.S.) | Ancillary |
| 170100000X | Ph.D. Medical Genetics | Ancillary |
| 101200000X | Drama Therapist | Ancillary |
| 367A00000X | Advanced Practice Midwife | APP |
| 364SX0204X | Pediatric Oncology Clinical Nurse Specialist | APP |
| 364SX0200X | Oncology Clinical Nurse Specialist | APP |
| 364SX0106X | Occupational Health Clinical Nurse Specialist | APP |
| 364SW0102X | Women's Health Clinical Nurse Specialist | APP |
| 364ST0500X | Transplantation Clinical Nurse Specialist | APP |
| 364SS0200X | School Clinical Nurse Specialist | APP |
| 364SR0400X | Rehabilitation Clinical Nurse Specialist | APP |
| 364SP1700X | Perinatal Clinical Nurse Specialist | APP |
| 364SP0813X | Geropsychiatric Psychiatric/Mental Health Clinical Nurse Specialist | APP |
| 364SP0812X | Community Psychiatric/Mental Health Clinical Nurse Specialist | APP |
| 364SP0811X | Chronically Ill Psychiatric/Mental Health Clinical Nurse Specialist | APP |
| 364SP0810X | Child & Family Psychiatric/Mental Health Clinical Nurse Specialist | APP |
| 364SP0809X | Adult Psychiatric/Mental Health Clinical Nurse Specialist | APP |
| 364SP0808X | Psychiatric/Mental Health Clinical Nurse Specialist | APP |
| 364SP0807X | Child & Adolescent Psychiatric/Mental Health Clinical Nurse Specialist | APP |
| 364SP0200X | Pediatric Clinical Nurse Specialist | APP |
| 364SN0800X | Neuroscience Clinical Nurse Specialist | APP |
| 364SN0000X | Neonatal Clinical Nurse Specialist | APP |
| 364SM0705X | Medical-Surgical Clinical Nurse Specialist | APP |
| 364SG0600X | Gerontology Clinical Nurse Specialist | APP |
| 364SF0001X | Family Health Clinical Nurse Specialist | APP |
| 364SE0003X | Emergency Clinical Nurse Specialist | APP |
| 364SC2300X | Chronic Care Clinical Nurse Specialist | APP |
| 364SC1501X | Community Health/Public Health Clinical Nurse Specialist | APP |
| 364SC0200X | Critical Care Medicine Clinical Nurse Specialist | APP |
| 364SA2200X | Adult Health Clinical Nurse Specialist | APP |
| 364SA2100X | Acute Care Clinical Nurse Specialist | APP |
| 364S00000X | Clinical Nurse Specialist | APP |
| 363LX0106X | Occupational Health Nurse Practitioner | APP |
| 363LX0001X | Obstetrics & Gynecology Nurse Practitioner | APP |
| 363LW0102X | Women's Health Nurse Practitioner | APP |
| 363LS0200X | School Nurse Practitioner | APP |
| 363LP2300X | Primary Care Nurse Practitioner | APP |
| 363LP1700X | Perinatal Nurse Practitioner | APP |
| 363LP0808X | Psychiatric/Mental Health Nurse Practitioner | APP |

© 2025 Hammond G et al. *JAMA Health Forum*.

| | | |
|---|---|---|
| 363LP0222X | Critical Care Pediatric Nurse Practitioner | APP |
| 363LP0200X | Pediatric Nurse Practitioner | APP |
| 363LN0005X | Critical Care Neonatal Nurse Practitioner | APP |
| 363LN0000X | Neonatal Nurse Practitioner | APP |
| 363LG0600X | Gerontology Nurse Practitioner | APP |
| 363LF0000X | Family Nurse Practitioner | APP |
| 363LC1500X | Community Health Nurse Practitioner | APP |
| 363LC0200X | Critical Care Medicine Nurse Practitioner | APP |
| 363LA2200X | Adult Health Nurse Practitioner | APP |
| 363LA2100X | Acute Care Nurse Practitioner | APP |
| 363L00000X | Nurse Practitioner | APP |
| 363AS0400X | Surgical Physician Assistant | APP |
| 363AM0700X | Medical Physician Assistant | APP |
| 363A00000X | Physician Assistant | APP |
| 176B00000X | Midwife | APP |
| 237600000X | Audiologist-Hearing Aid Fitter | Audiology |
| 231HA2500X | Assistive Technology Supplier Audiologist | Audiology |
| 231HA2400X | Assistive Technology Practitioner Audiologist | Audiology |
| 231H00000X | Audiologist | Audiology |
| 171M00000X | Case Manager/Care Coordinator | Case Manager |
| 111NX0800X | Orthopedic Chiropractor | Chiropractor |
| 111NX0100X | Occupational Health Chiropractor | Chiropractor |
| 111NS0005X | Sports Physician Chiropractor | Chiropractor |
| 111NR0400X | Rehabilitation Chiropractor | Chiropractor |
| 111NR0200X | Radiology Chiropractor | Chiropractor |
| 111NN1001X | Nutrition Chiropractor | Chiropractor |
| 111NN0400X | Neurology Chiropractor | Chiropractor |
| 111NI0900X | Internist Chiropractor | Chiropractor |
| 111NI0013X | Independent Medical Examiner Chiropractor | Chiropractor |
| 111N00000X | Chiropractor | Chiropractor |
| 324500000X | Substance Abuse Rehabilitation Facility | Clinic |
| 261QX0203X | Radiation Oncology Clinic/Center | Clinic |
| 261QX0200X | Oncology Clinic/Center | Clinic |
| 261QX0100X | Occupational Medicine Clinic/Center | Clinic |
| 261QV0200X | VA Clinic/Center | Clinic |
| 261QU0200X | Urgent Care Clinic/Center | Clinic |
| 261QS1200X | Sleep Disorder Diagnostic Clinic/Center | Clinic |
| 261QS1000X | Student Health Clinic/Center | Clinic |
| 261QS0132X | Ophthalmologic Surgery Clinic/Center | Clinic |
| 261QR1100X | Research Clinic/Center | Clinic |

| | | |
|---|---|---|
| 261QR0405X | Substance Use Disorder Rehabilitation Clinic/Center | Clinic |
| 261QR0400X | Rehabilitation Clinic/Center | Clinic |
| 261QR0208X | Mobile Radiology Clinic/Center | Clinic |
| 261QR0207X | Mobile Mammography Clinic/Center | Clinic |
| 261QR0206X | Mammography Clinic/Center | Clinic |
| 261QR0200X | Radiology Clinic/Center | Clinic |
| 261QP3300X | Pain Clinic/Center | Clinic |
| 261QP2300X | Primary Care Clinic/Center | Clinic |
| 261QP2000X | Physical Therapy Clinic/Center | Clinic |
| 261QP1100X | Podiatric Clinic/Center | Clinic |
| 261QM2500X | Medical Specialty Clinic/Center | Clinic |
| 261QM1300X | Multi-Specialty Clinic/Center | Clinic |
| 261QM1200X | Magnetic Resonance Imaging (MRI) Clinic/Center | Clinic |
| 261QM0855X | Adolescent and Children Mental Health Clinic/Center | Clinic |
| 261QM0850X | Adult Mental Health Clinic/Center | Clinic |
| 261QM0801X | Mental Health Clinic/Center (Including Community Mental Health Center) | Clinic |
| 261QI0500X | Infusion Therapy Clinic/Center | Clinic |
| 261QH0700X | Hearing and Speech Clinic/Center | Clinic |
| 261QH0100X | Health Service Clinic/Center | Clinic |
| 261QE0800X | Endoscopy Clinic/Center | Clinic |
| 261QE0002X | Emergency Care Clinic/Center | Clinic |
| 261QD0000X | Dental Clinic/Center | Clinic |
| 261QC1800X | Corporate Health Clinic/Center | Clinic |
| 261QC1500X | Community Health Clinic/Center | Clinic |
| 261QA1903X | Ambulatory Surgical Clinic/Center | Clinic |
| 261QA0006X | Ambulatory Fertility Facility | Clinic |
| 261QA0005X | Ambulatory Family Planning Facility | Clinic |
| 261Q00000X | Clinic/Center | Clinic |
| 193400000X | Single Specialty Group | Clinic |
| 193200000X | Multi-Specialty Group | Clinic |
| 171000000X | Military Health Care Provider | Clinic |
| 172V00000X | Community Health Worker | Community Health Worker |
| 282NC2000X | Children's Hospital | Critical Access Hospital |
| 282NC0060X | Critical Access Hospital | Critical Access Hospital |
| 261QC0050X | Critical Access Hospital Clinic/Center | Critical Access Hospital |
| 125Q00000X | Oral Medicinist | Dental |
| 125K00000X | Advanced Practice Dental Therapist | Dental |
| 124Q00000X | Dental Hygienist | Dental |

© 2025 Hammond G et al. *JAMA Health Forum*.

| | | |
|---|---|---|
| 1223X2210X | Orofacial Pain Dentist | Dental |
| 1223X0400X | Orthodontics and Dentofacial Orthopedic Dentist | Dental |
| 1223X0008X | Oral and Maxillofacial Radiology Dentist | Dental |
| 1223S0112X | Oral and Maxillofacial Surgery (Dentist) | Dental |
| 1223P0221X | Pediatric Dentist | Dental |
| 1223P0106X | Oral and Maxillofacial Pathology Dentist | Dental |
| 1223G0001X | General Practice Dentistry | Dental |
| 1223E0200X | Endodontist | Dental |
| 1223D0001X | Public Health Dentist | Dental |
| 122300000X | Dentist | Dental |
| 1223P0700X | Prosthodontist | Dental |
| 1223P0300X | Periodontist | Dental |
| 1223D0004X | Dentist Anesthesiologist | Dental |
| 133VN1501X | Sports Dietetics Nutrition Registered Dietitian | Dietician |
| 133VN1401X | Pediatric Critical Care Nutrition Registered Dietitian | Dietician |
| 133VN1301X | Oncology Nutrition Registered Dietitian | Dietician |
| 133VN1101X | Gerontological Nutrition Registered Dietitian | Dietician |
| 132700000X | Dietary Manager | Dietician |
| 133VN1201X | Obesity and Weight Management Nutrition Registered Dietitian | Dietician/Nutrition |
| 133VN1006X | Metabolic Nutrition Registered Dietitian | Dietician/Nutrition |
| 133VN1005X | Renal Nutrition Registered Dietitian | Dietician/Nutrition |
| 133VN1004X | Pediatric Nutrition Registered Dietitian | Dietician/Nutrition |
| 133V00000X | Registered Dietitian | Dietician/Nutrition |
| 133NN1002X | Nutrition Education Nutritionist | Dietician/Nutrition |
| 133N00000X | Nutritionist | Dietician/Nutrition |
| 335V00000X | Portable X-ray and/or Other Portable Diagnostic Imaging Supplier | DME |
| 335E00000X | Prosthetic/Orthotic Supplier | DME |
| 332H00000X | Eyewear Supplier | DME |
| 332BX2000X | Oxygen Equipment & Supplies (DME) | DME |
| 332BP3500X | Parenteral & Enteral Nutrition Supplies (DME) | DME |
| 332BN1400X | Nursing Facility Supplies (DME) | DME |
| 332BC3200X | Customized Equipment (DME) | DME |
| 332B00000X | Durable Medical Equipment & Medical Supplies | DME |
| 2080P0204X | Pediatric Emergency Medicine (Pediatrics) Physician | Emergency |
| 207XX0801X | Orthopaedic Trauma Physician | Emergency |
| 207PS0010X | Sports Medicine (Emergency Medicine) Physician | Emergency |
| 207PP0204X | Pediatric Emergency Medicine (Emergency Medicine) Physician | Emergency |
| 207PH0002X | Hospice and Palliative Medicine (Emergency Medicine) Physician | Emergency |
| 207PE0005X | Undersea and Hyperbaric Medicine (Emergency Medicine) Physician | Emergency |
| 207PE0004X | Emergency Medical Services (Emergency Medicine) Physician | Emergency |

© 2025 Hammond G et al. *JAMA Health Forum*.

| | | |
|---|---|---|
| 207P00000X | Emergency Medicine Physician | Emergency |
| 261QE0700X | End-Stage Renal Disease (ESRD) Treatment Clinic/Center | ESRD |
| 261QP0905X | State or Local Public Health Clinic/Center | FQHC/Critical Access Clinic |
| 261QP0904X | Federal Public Health Clinic/Center | FQHC/Critical Access Clinic |
| 261QF0400X | Federally Qualified Health Center (FQHC) | FQHC/Critical Access Clinic |
| 207RG0300X | Geriatric Medicine (Internal Medicine) Physician | Geriatrics |
| 207QG0300X | Geriatric Medicine (Family Medicine) Physician | Geriatrics |
| 253Z00000X | In Home Supportive Care Agency | Home Health Org |
| 251E00000X | Home Health Agency | Home Health Org |
| 2085H0002X | Hospice and Palliative Medicine (Radiology) Physician | Hospice |
| 2084H0002X | Hospice and Palliative Medicine (Psychiatry & Neurology) Physician | Hospice |
| 2081H0002X | Hospice and Palliative Medicine (Physical Medicine & Rehabilitation) Physician | Hospice |
| 2080H0002X | Pediatric Hospice and Palliative Medicine Physician | Hospice |
| 207VH0002X | Hospice and Palliative Medicine (Obstetrics & Gynecology) Physician | Hospice |
| 207RH0002X | Hospice and Palliative Medicine (Internal Medicine) Physician | Hospice |
| 207QH0002X | Hospice and Palliative Medicine (Family Medicine) Physician | Hospice |
| 207LH0002X | Hospice and Palliative Medicine (Anesthesiology) Physician | Hospice |
| 315D00000X | Inpatient Hospice | Hospice Org |
| 251G00000X | Community Based Hospice Care Agency | Hospice Org |
| 2865M2000X | Military General Acute Care Hospital | Hospital |
| 286500000X | Military Hospital | Hospital |
| 284300000X | Special Hospital | Hospital |
| 283XC2000X | Children's Rehabilitation Hospital | Hospital |
| 283X00000X | Rehabilitation Hospital | Hospital |
| 283Q00000X | Psychiatric Hospital | Hospital |
| 282NW0100X | Women's Hospital | Hospital |
| 282N00000X | General Acute Care Hospital | Hospital |
| 282E00000X | Long Term Care Hospital | Hospital |
| 281PC2000X | Children' s Chronic Disease Hospital | Hospital |
| 281P00000X | Chronic Disease Hospital | Hospital |
| 276400000X | Substance Use Disorder Rehabilitation Hospital Unit | Hospital |
| 275N00000X | Medicare Defined Swing Bed Hospital Unit | Hospital |
| 273Y00000X | Rehabilitation Hospital Unit | Hospital |
| 273R00000X | Psychiatric Hospital Unit | Hospital |
| 273100000X | Epilepsy Hospital Unit | Hospital |
| 208M00000X | Hospitalist Physician | Hospitalist |
| 293D00000X | Physiological Laboratory | Lab |

© 2025 Hammond G et al. *JAMA Health Forum*.

| | | |
|---|---|---|
| 291U00000X | Clinical Medical Laboratory | Lab |
| 385HR2055X | Child Mental Illness Respite Care | LTC |
| 385H00000X | Respite Care | LTC |
| 320800000X | Mental Illness Community Based Residential Treatment Facility | LTC |
| 320700000X | Physical Disabilities Residential Treatment Facility | LTC |
| 320600000X | Intellectual and/or Developmental Disabilities Residential Treatment Facility | LTC |
| 3140N1450X | Pediatric Skilled Nursing Facility | LTC |
| 314000000X | Skilled Nursing Facility | LTC |
| 313M00000X | Nursing Facility/Intermediate Care Facility | LTC |
| 311ZA0620X | Adult Care Home Facility | LTC |
| 311Z00000X | Custodial Care Facility | LTC |
| 311500000X | Alzheimer Center (Dementia Center) | LTC |
| 310400000X | Assisted Living Facility | LTC |
| 261QA0600X | Adult Day Care Clinic/Center | LTC |
| 305S00000X | Point of Service | Managed Care |
| 305R00000X | Preferred Provider Organization | Managed Care |
| 302R00000X | Health Maintenance Organization | Managed Care |
| 302F00000X | Exclusive Provider Organization | Managed Care |
| 251T00000X | PACE Provider Organization | Managed Care |
| 251B00000X | Case Management Agency | Managed Care |
| 2084P0805X | Geriatric Psychiatry Physician | Mental Health |
| 2084P0804X | Child & Adolescent Psychiatry Physician | Mental Health |
| 2084P0802X | Addiction Psychiatry Physician | Mental Health |
| 2084P0800X | Psychiatry Physician | Mental Health |
| 2084F0202X | Forensic Psychiatry Physician | Mental Health |
| 2084B0040X | Behavioral Neurology & Neuropsychiatry Physician | Mental Health |
| 2084A0401X | Addiction Medicine (Psychiatry & Neurology) Physician | Mental Health |
| 2083A0300X | Addiction Medicine (Preventive Medicine) Physician | Mental Health |
| 207RA0401X | Addiction Medicine (Internal Medicine) Physician | Mental Health |
| 175T00000X | Peer Specialist | MSW/Psychology |
| 171400000X | Health & Wellness Coach | MSW/Psychology |
| 163WA0400X | Addiction (Substance Use Disorder) Registered Nurse | MSW/Psychology |
| 106H00000X | Marriage & Family Therapist | MSW/Psychology |
| 1041S0200X | School Social Worker | MSW/Psychology |
| 1041C0700X | Clinical Social Worker | MSW/Psychology |
| 104100000X | Social Worker | MSW/Psychology |
| 103TS0200X | School Psychologist | MSW/Psychology |
| 103TR0400X | Rehabilitation Psychologist | MSW/Psychology |
| 103TP2701X | Group Psychotherapy Psychologist | MSW/Psychology |
| 103TP0814X | Psychoanalysis Psychologist | MSW/Psychology |

© 2025 Hammond G et al. *JAMA Health Forum*.

| | | |
|---|---|---|
| 103TP0016X | Prescribing (Medical) Psychologist | MSW/Psychology |
| 103TM1800X | Intellectual & Developmental Disabilities Psychologist | MSW/Psychology |
| 103TH0100X | Health Service Psychologist | MSW/Psychology |
| 103TH0004X | Health Psychologist | MSW/Psychology |
| 103TF0200X | Forensic Psychologist | MSW/Psychology |
| 103TF0000X | Family Psychologist | MSW/Psychology |
| 103TE1100X | Exercise & Sports Psychologist | MSW/Psychology |
| 103TC2200X | Clinical Child & Adolescent Psychologist | MSW/Psychology |
| 103TC1900X | Counseling Psychologist | MSW/Psychology |
| 103TC0700X | Clinical Psychologist | MSW/Psychology |
| 103TB0200X | Cognitive & Behavioral Psychologist | MSW/Psychology |
| 103TA0700X | Adult Development & Aging Psychologist | MSW/Psychology |
| 103TA0400X | Addiction (Substance Use Disorder) Psychologist | MSW/Psychology |
| 103T00000X | Psychologist | MSW/Psychology |
| 103K00000X | Behavioral Analyst | MSW/Psychology |
| 103G00000X | Clinical Neuropsychologist | MSW/Psychology |
| 102L00000X | Psychoanalyst | MSW/Psychology |
| 101YS0200X | School Counselor | MSW/Psychology |
| 101YP2500X | Professional Counselor | MSW/Psychology |
| 101YP1600X | Pastoral Counselor | MSW/Psychology |
| 101YM0800X | Mental Health Counselor | MSW/Psychology |
| 101YA0400X | Addiction (Substance Use Disorder) Counselor | MSW/Psychology |
| 101Y00000X | Counselor | MSW/Psychology |
| 376K00000X | Nurse's Aide | Nurse/Assistant |
| 374U00000X | Home Health Aide | Nurse/Assistant |
| 374700000X | Technician | Nurse/Assistant |
| 372500000X | Chore Provider | Nurse/Assistant |
| 367H00000X | Anesthesiologist Assistant | Nurse/Assistant |
| 367500000X | Certified Registered Nurse Anesthetist | Nurse/Assistant |
| 247200000X | Other Technician | Nurse/Assistant |
| 2471S1302X | Sonography Radiologic Technologist | Nurse/Assistant |
| 2471R0002X | Radiation Therapy Radiologic Technologist | Nurse/Assistant |
| 2471N0900X | Nuclear Medicine Technology Radiologic Technologist | Nurse/Assistant |
| 2471M1202X | Magnetic Resonance Imaging Radiologic Technologist | Nurse/Assistant |
| 2471C3402X | Radiography Radiologic Technologist | Nurse/Assistant |
| 2471C3401X | Computed Tomography Radiologic Technologist | Nurse/Assistant |
| 2471C1106X | Cardiac-Interventional Technology Radiologic Technologist | Nurse/Assistant |
| 2471B0102X | Bone Densitometry Radiologic Technologist | Nurse/Assistant |
| 247100000X | Radiologic Technologist | Nurse/Assistant |
| 246ZX2200X | Orthopedic Assistant | Nurse/Assistant |
| 246ZS0410X | Surgical Technologist | Nurse/Assistant |

© 2025 Hammond G et al. *JAMA Health Forum*.

| | | |
|---|---|---|
| 246ZN0300X | Nephrology Specialist/Technologist | Nurse/Assistant |
| 246ZE0600X | Electroneurodiagnostic Specialist/Technologist | Nurse/Assistant |
| 246ZC0007X | Surgical Assistant | Nurse/Assistant |
| 246Z00000X | Other Specialist/Technologist | Nurse/Assistant |
| 246XC2903X | Vascular Specialist/Technologist | Nurse/Assistant |
| 246X00000X | Cardiovascular Specialist/Technologist | Nurse/Assistant |
| 246W00000X | Cardiology Technician | Nurse/Assistant |
| 246RP1900X | Phlebotomy Technician | Nurse/Assistant |
| 246QM0706X | Medical Technologist | Nurse/Assistant |
| 246QH0600X | Histology Specialist/Technologist | Nurse/Assistant |
| 246Q00000X | Pathology Specialist/Technologist | Nurse/Assistant |
| 243U00000X | Radiology Practitioner Assistant | Nurse/Assistant |
| 2355S0801X | Speech-Language Assistant | Nurse/Assistant |
| 2355A2700X | Audiology Assistant | Nurse/Assistant |
| 225500000X | Respiratory/Developmental/Rehabilitative Specialist/Technologist | Nurse/Assistant |
| 225200000X | Physical Therapy Assistant | Nurse/Assistant |
| 224Z00000X | Occupational Therapy Assistant | Nurse/Assistant |
| 211D00000X | Podiatric Assistant | Nurse/Assistant |
| 183700000X | Pharmacy Technician | Nurse/Assistant |
| 175M00000X | Lay Midwife | Nurse/Assistant |
| 1710I1003X | Independent Duty Medical Technicians | Nurse/Assistant |
| 1710I1002X | Independent Duty Corpsman | Nurse/Assistant |
| 164X00000X | Licensed Vocational Nurse | Nurse/Assistant |
| 164W00000X | Licensed Practical Nurse | Nurse/Assistant |
| 163WX0800X | Orthopedic Registered Nurse | Nurse/Assistant |
| 163WX0601X | Otorhinolaryngology & Head-Neck Registered Nurse | Nurse/Assistant |
| 163WX0200X | Oncology Registered Nurse | Nurse/Assistant |
| 163WX0106X | Occupational Health Registered Nurse | Nurse/Assistant |
| 163WX0003X | Inpatient Obstetric Registered Nurse | Nurse/Assistant |
| 163WX0002X | High-Risk Obstetric Registered Nurse | Nurse/Assistant |
| 163WW0101X | Ambulatory Women's Health Care Registered Nurse | Nurse/Assistant |
| 163WW0000X | Wound Care Registered Nurse | Nurse/Assistant |
| 163WU0100X | Urology Registered Nurse | Nurse/Assistant |
| 163WS0200X | School Registered Nurse | Nurse/Assistant |
| 163WS0121X | Plastic Surgery Registered Nurse | Nurse/Assistant |
| 163WR1000X | Reproductive Endocrinology/Infertility Registered Nurse | Nurse/Assistant |
| 163WR0400X | Rehabilitation Registered Nurse | Nurse/Assistant |
| 163WR0006X | Registered Nurse First Assistant | Nurse/Assistant |
| 163WP2201X | Ambulatory Care Registered Nurse | Nurse/Assistant |
| 163WP1700X | Perinatal Registered Nurse | Nurse/Assistant |

© 2025 Hammond G et al. *JAMA Health Forum*.

| | | |
|---|---|---|
| 163WP0809X | Adult Psychiatric/Mental Health Registered Nurse | Nurse/Assistant |
| 163WP0808X | Psychiatric/Mental Health Registered Nurse | Nurse/Assistant |
| 163WP0807X | Child & Adolescent Psychiatric/Mental Health Registered Nurse | Nurse/Assistant |
| 163WP0218X | Pediatric Oncology Registered Nurse | Nurse/Assistant |
| 163WP0200X | Pediatric Registered Nurse | Nurse/Assistant |
| 163WP0000X | Pain Management Registered Nurse | Nurse/Assistant |
| 163WN1003X | Nutrition Support Registered Nurse | Nurse/Assistant |
| 163WN0800X | Neuroscience Registered Nurse | Nurse/Assistant |
| 163WN0300X | Nephrology Registered Nurse | Nurse/Assistant |
| 163WN0003X | Low-Risk Neonatal Registered Nurse | Nurse/Assistant |
| 163WN0002X | Neonatal Intensive Care Registered Nurse | Nurse/Assistant |
| 163WM0705X | Medical-Surgical Registered Nurse | Nurse/Assistant |
| 163WM0102X | Maternal Newborn Registered Nurse | Nurse/Assistant |
| 163WL0100X | Lactation Consultant (Registered Nurse) | Nurse/Assistant |
| 163WI0600X | Infection Control Registered Nurse | Nurse/Assistant |
| 163WI0500X | Infusion Therapy Registered Nurse | Nurse/Assistant |
| 163WH1000X | Hospice Registered Nurse | Nurse/Assistant |
| 163WH0500X | Hemodialysis Registered Nurse | Nurse/Assistant |
| 163WH0200X | Home Health Registered Nurse | Nurse/Assistant |
| 163WG0600X | Gerontology Registered Nurse | Nurse/Assistant |
| 163WG0100X | Gastroenterology Registered Nurse | Nurse/Assistant |
| 163WG0000X | General Practice Registered Nurse | Nurse/Assistant |
| 163WF0300X | Flight Registered Nurse | Nurse/Assistant |
| 163WE0900X | Enterostomal Therapy Registered Nurse | Nurse/Assistant |
| 163WE0003X | Emergency Registered Nurse | Nurse/Assistant |
| 163WD1100X | Peritoneal Dialysis Registered Nurse | Nurse/Assistant |
| 163WD0400X | Diabetes Educator Registered Nurse | Nurse/Assistant |
| 163WC3500X | Cardiac Rehabilitation Registered Nurse | Nurse/Assistant |
| 163WC2100X | Continence Care Registered Nurse | Nurse/Assistant |
| 163WC1600X | Continuing Education/Staff Development Registered Nurse | Nurse/Assistant |
| 163WC1500X | Community Health Registered Nurse | Nurse/Assistant |
| 163WC1400X | College Health Registered Nurse | Nurse/Assistant |
| 163WC0400X | Case Management Registered Nurse | Nurse/Assistant |
| 163WC0200X | Critical Care Medicine Registered Nurse | Nurse/Assistant |
| 163WA2000X | Administrator Registered Nurse | Nurse/Assistant |
| 163W00000X | Registered Nurse | Nurse/Assistant |
| 156FX1101X | Ophthalmic Assistant | Nurse/Assistant |
| 146N00000X | Basic Emergency Medical Technician | Nurse/Assistant |
| 146M00000X | Intermediate Emergency Medical Technician | Nurse/Assistant |
| 146L00000X | Paramedic | Nurse/Assistant |

© 2025 Hammond G et al. *JAMA Health Forum*.

| | | |
|---|---|---|
| 146D00000X | Personal Emergency Response Attendant | Nurse/Assistant |
| 136A00000X | Registered Dietetic Technician | Nurse/Assistant |
| 106S00000X | Behavior Technician | Nurse/Assistant |
| 251J00000X | Nursing Care Agency | Nursing Care |
| 152WV0400X | Vision Therapy Optometrist | Optometry |
| 152WS0006X | Sports Vision Optometrist | Optometry |
| 152WP0200X | Pediatric Optometrist | Optometry |
| 152WL0500X | Low Vision Rehabilitation Optometrist | Optometry |
| 152WC0802X | Corneal and Contact Management Optometrist | Optometry |
| 152W00000X | Optometrist | Optometry |
| 347C00000X | Private Vehicle | Other Org |
| 343900000X | Non-emergency Medical Transport (VAN) | Other Org |
| 3416L0300X | Land Ambulance | Other Org |
| 3416A0800X | Air Ambulance | Other Org |
| 341600000X | Ambulance | Other Org |
| 335G00000X | Medical Foods Supplier | Other Org |
| 332U00000X | Home Delivered Meals | Other Org |
| 261QR0401X | Comprehensive Outpatient Rehabilitation Facility (CORF) | Other Org |
| 252Y00000X | Early Intervention Provider Agency | Other Org |
| 251X00000X | Supports Brokerage Agency | Other Org |
| 251V00000X | Voluntary or Charitable Agency | Other Org |
| 251S00000X | Community/Behavioral Health Agency | Other Org |
| 251K00000X | Public Health or Welfare Agency | Other Org |
| 251F00000X | Home Infusion Agency | Other Org |
| 251C00000X | Developmentally Disabled Services Day Training Agency | Other Org |
| 209800000X | Legal Medicine (M.D./D.O.) Physician | Other Specialty |
| 208VP0014X | Interventional Pain Medicine Physician | Other Specialty |
| 208VP0000X | Pain Medicine Physician | Other Specialty |
| 208U00000X | Clinical Pharmacology Physician | Other Specialty |
| 208G00000X | Thoracic Surgery (Cardiothoracic Vascular Surgery) Physician | Other Specialty |
| 2088P0231X | Pediatric Urology Physician | Other Specialty |
| 2088F0040X | Female Pelvic Medicine and Reconstructive Surgery (Urology) Physician | Other Specialty |
| 208800000X | Urology Physician | Other Specialty |
| 2085U0001X | Diagnostic Ultrasound Physician | Other Specialty |
| 2085R0205X | Radiological Physics Physician | Other Specialty |
| 2085R0204X | Vascular & Interventional Radiology Physician | Other Specialty |
| 2085R0203X | Therapeutic Radiology Physician | Other Specialty |
| 2085R0202X | Diagnostic Radiology Physician | Other Specialty |
| 2085R0001X | Radiation Oncology Physician | Other Specialty |
| 2085P0229X | Pediatric Radiology Physician | Other Specialty |

© 2025 Hammond G et al. *JAMA Health Forum*.

| | | |
|---|---|---|
| 2085N0904X | Nuclear Radiology Physician | Other Specialty |
| 2085N0700X | Neuroradiology Physician | Other Specialty |
| 2085D0003X | Diagnostic Neuroimaging (Radiology) Physician | Other Specialty |
| 2085B0100X | Body Imaging Physician | Other Specialty |
| 2084V0102X | Vascular Neurology Physician | Other Specialty |
| 2084S0012X | Sleep Medicine (Psychiatry & Neurology) Physician | Other Specialty |
| 2084S0010X | Sports Medicine (Psychiatry & Neurology) Physician | Other Specialty |
| 2084P2900X | Pain Medicine (Psychiatry & Neurology) Physician | Other Specialty |
| 2084P0301X | Brain Injury Medicine (Psychiatry & Neurology) Physician | Other Specialty |
| 2084P0015X | Psychosomatic Medicine Physician | Other Specialty |
| 2084P0005X | Neurodevelopmental Disabilities Physician | Other Specialty |
| 2084N0600X | Clinical Neurophysiology Physician | Other Specialty |
| 2084N0402X | Neurology with Special Qualifications in Child Neurology Physician | Other Specialty |
| 2084N0400X | Neurology Physician | Other Specialty |
| 2084N0008X | Neuromuscular Medicine (Psychiatry & Neurology) Physician | Other Specialty |
| 2084E0001X | Epilepsy Physician | Other Specialty |
| 2084D0003X | Diagnostic Neuroimaging (Psychiatry & Neurology) Physician | Other Specialty |
| 2084B0002X | Obesity Medicine (Psychiatry & Neurology) Physician | Other Specialty |
| 2084A2900X | Neurocritical Care Physician | Other Specialty |
| 2083X0100X | Occupational Medicine Physician | Other Specialty |
| 2083T0002X | Medical Toxicology (Preventive Medicine) Physician | Other Specialty |
| 2083S0010X | Sports Medicine (Preventive Medicine) Physician | Other Specialty |
| 2083P0011X | Undersea and Hyperbaric Medicine (Preventive Medicine) Physician | Other Specialty |
| 2083C0008X | Clinical Informatics Physician | Other Specialty |
| 2083A0100X | Aerospace Medicine Physician | Other Specialty |
| 2081S0010X | Sports Medicine (Physical Medicine & Rehabilitation) Physician | Other Specialty |
| 2081P2900X | Pain Medicine (Physical Medicine & Rehabilitation) Physician | Other Specialty |
| 2081P0301X | Brain Injury Medicine (Physical Medicine & Rehabilitation) Physician | Other Specialty |
| 2081P0010X | Pediatric Rehabilitation Medicine Physician | Other Specialty |
| 2081P0004X | Spinal Cord Injury Medicine Physician | Other Specialty |
| 2081N0008X | Neuromuscular Medicine (Physical Medicine & Rehabilitation) Physician | Other Specialty |
| 208100000X | Physical Medicine & Rehabilitation Physician | Other Specialty |
| 2080T0004X | Pediatric Transplant Hepatology Physician | Other Specialty |
| 2080T0002X | Pediatric Medical Toxicology Physician | Other Specialty |
| 2080S0012X | Pediatric Sleep Medicine Physician | Other Specialty |
| 2080S0010X | Pediatric Sports Medicine Physician | Other Specialty |
| 2080P0216X | Pediatric Rheumatology Physician | Other Specialty |
| 2080P0214X | Pediatric Pulmonology Physician | Other Specialty |
| 2080P0210X | Pediatric Nephrology Physician | Other Specialty |

© 2025 Hammond G et al. *JAMA Health Forum*.

| | | |
|---|---|---|
| 2080P0208X | Pediatric Infectious Diseases Physician | Other Specialty |
| 2080P0207X | Pediatric Hematology & Oncology Physician | Other Specialty |
| 2080P0206X | Pediatric Gastroenterology Physician | Other Specialty |
| 2080P0205X | Pediatric Endocrinology Physician | Other Specialty |
| 2080P0203X | Pediatric Critical Care Medicine Physician | Other Specialty |
| 2080P0202X | Pediatric Cardiology Physician | Other Specialty |
| 2080P0201X | Pediatric Allergy/Immunology Physician | Other Specialty |
| 2080P0008X | Pediatric Neurodevelopmental Disabilities Physician | Other Specialty |
| 2080P0006X | Developmental - Behavioral Pediatrics Physician | Other Specialty |
| 2080N0001X | Neonatal-Perinatal Medicine Physician | Other Specialty |
| 2080I0007X | Pediatric Clinical & Laboratory Immunology Physician | Other Specialty |
| 2080C0008X | Child Abuse Pediatrics Physician | Other Specialty |
| 2080B0002X | Pediatric Obesity Medicine Physician | Other Specialty |
| 2080A0000X | Pediatric Adolescent Medicine Physician | Other Specialty |
| 207ZP0213X | Pediatric Pathology Physician | Other Specialty |
| 207ZP0105X | Clinical Pathology/Laboratory Medicine Physician | Other Specialty |
| 207ZP0104X | Chemical Pathology Physician | Other Specialty |
| 207ZP0102X | Anatomic Pathology & Clinical Pathology Physician | Other Specialty |
| 207ZP0101X | Anatomic Pathology Physician | Other Specialty |
| 207ZP0007X | Molecular Genetic Pathology (Pathology) Physician | Other Specialty |
| 207ZN0500X | Neuropathology Physician | Other Specialty |
| 207ZM0300X | Medical Microbiology Physician | Other Specialty |
| 207ZI0100X | Immunopathology Physician | Other Specialty |
| 207ZH0000X | Hematology (Pathology) Physician | Other Specialty |
| 207ZF0201X | Forensic Pathology Physician | Other Specialty |
| 207ZD0900X | Dermatopathology (Pathology) Physician | Other Specialty |
| 207ZC0500X | Cytopathology Physician | Other Specialty |
| 207ZC0008X | Clinical Informatics (Pathology) Physician | Other Specialty |
| 207ZC0006X | Clinical Pathology Physician | Other Specialty |
| 207ZB0001X | Blood Banking & Transfusion Medicine Physician | Other Specialty |
| 207YX0905X | Otolaryngology/Facial Plastic Surgery Physician | Other Specialty |
| 207YX0901X | Otology & Neurotology Physician | Other Specialty |
| 207YX0602X | Otolaryngic Allergy Physician | Other Specialty |
| 207YX0007X | Plastic Surgery within the Head & Neck (Otolaryngology) Physician | Other Specialty |
| 207YS0012X | Sleep Medicine (Otolaryngology) Physician | Other Specialty |
| 207YP0228X | Pediatric Otolaryngology Physician | Other Specialty |
| 207Y00000X | Otolaryngology Physician | Other Specialty |
| 207XX0005X | Sports Medicine (Orthopaedic Surgery) Physician | Other Specialty |
| 207XS0114X | Adult Reconstructive Orthopaedic Surgery Physician | Other Specialty |
| 207XP3100X | Pediatric Orthopaedic Surgery Physician | Other Specialty |

© 2025 Hammond G et al. *JAMA Health Forum*.

| | | |
|---|---|---|
| 207WX0200X | Ophthalmic Plastic and Reconstructive Surgery Physician | Other Specialty |
| 207WX0120X | Cornea and External Diseases Specialist Physician | Other Specialty |
| 207WX0110X | Pediatric Ophthalmology and Strabismus Specialist Physician Physician | Other Specialty |
| 207WX0109X | Neuro-ophthalmology Physician | Other Specialty |
| 207WX0108X | Uveitis and Ocular Inflammatory Disease (Ophthalmology) Physician | Other Specialty |
| 207WX0107X | Retina Specialist (Ophthalmology) Physician | Other Specialty |
| 207WX0009X | Glaucoma Specialist (Ophthalmology) Physician | Other Specialty |
| 207W00000X | Ophthalmology Physician | Other Specialty |
| 207VX0201X | Gynecologic Oncology Physician | Other Specialty |
| 207VM0101X | Maternal & Fetal Medicine Physician | Other Specialty |
| 207VF0040X | Female Pelvic Medicine and Reconstructive Surgery (Obstetrics & Gynecology) Physician | Other Specialty |
| 207VE0102X | Reproductive Endocrinology Physician | Other Specialty |
| 207VC0300X | Complex Family Planning Physician | Other Specialty |
| 207VC0200X | Critical Care Medicine (Obstetrics & Gynecology) Physician | Other Specialty |
| 207VB0002X | Obesity Medicine (Obstetrics & Gynecology) Physician | Other Specialty |
| 207UN0903X | In Vivo & In Vitro Nuclear Medicine Physician | Other Specialty |
| 207UN0902X | Nuclear Imaging & Therapy Physician | Other Specialty |
| 207UN0901X | Nuclear Cardiology Physician | Other Specialty |
| 207U00000X | Nuclear Medicine Physician | Other Specialty |
| 207T00000X | Neurological Surgery Physician | Other Specialty |
| 207SM0001X | Molecular Genetic Pathology (Medical Genetics) Physician | Other Specialty |
| 207SG0205X | Ph.D. Medical Genetics Physician | Other Specialty |
| 207SG0203X | Clinical Molecular Genetics Physician | Other Specialty |
| 207SG0202X | Clinical Biochemical Genetics Physician | Other Specialty |
| 207SG0201X | Clinical Genetics (M.D.) Physician | Other Specialty |
| 207SC0300X | Clinical Cytogenetics Physician | Other Specialty |
| 207RX0202X | Medical Oncology Physician | Other Specialty |
| 207RT0003X | Transplant Hepatology Physician | Other Specialty |
| 207RS0012X | Sleep Medicine (Internal Medicine) Physician | Other Specialty |
| 207RS0010X | Sports Medicine (Internal Medicine) Physician | Other Specialty |
| 207RR0500X | Rheumatology Physician | Other Specialty |
| 207RP1001X | Pulmonary Disease Physician | Other Specialty |
| 207RN0300X | Nephrology Physician | Other Specialty |
| 207RM1200X | Magnetic Resonance Imaging (MRI) Internal Medicine Physician | Other Specialty |
| 207RI0200X | Infectious Disease Physician | Other Specialty |
| 207RI0011X | Interventional Cardiology Physician | Other Specialty |
| 207RI0008X | Hepatology Physician | Other Specialty |
| 207RI0001X | Clinical & Laboratory Immunology (Internal Medicine) Physician | Other Specialty |
| 207RH0005X | Hypertension Specialist Physician | Other Specialty |

© 2025 Hammond G et al. *JAMA Health Forum*.

| | | |
|---|---|---|
| 207RH0003X | Hematology & Oncology Physician | Other Specialty |
| 207RH0000X | Hematology (Internal Medicine) Physician | Other Specialty |
| 207RG0100X | Gastroenterology Physician | Other Specialty |
| 207RE0101X | Endocrinology, Diabetes & Metabolism Physician | Other Specialty |
| 207RC0200X | Critical Care Medicine (Internal Medicine) Physician | Other Specialty |
| 207RC0001X | Clinical Cardiac Electrophysiology Physician | Other Specialty |
| 207RC0000X | Cardiovascular Disease Physician | Other Specialty |
| 207RB0002X | Obesity Medicine (Internal Medicine) Physician | Other Specialty |
| 207RA0201X | Allergy & Immunology (Internal Medicine) Physician | Other Specialty |
| 207RA0002X | Adult Congenital Heart Disease Physician | Other Specialty |
| 207RA0001X | Advanced Heart Failure and Transplant Cardiology Physician | Other Specialty |
| 207RA0000X | Adolescent Medicine (Internal Medicine) Physician | Other Specialty |
| 207QS1201X | Sleep Medicine (Family Medicine) Physician | Other Specialty |
| 207QS0010X | Sports Medicine (Family Medicine) Physician | Other Specialty |
| 207QA0401X | Addiction Medicine (Family Medicine) Physician | Other Specialty |
| 207PT0002X | Medical Toxicology (Emergency Medicine) Physician | Other Specialty |
| 207NS0135X | Procedural Dermatology Physician | Other Specialty |
| 207NP0225X | Pediatric Dermatology Physician | Other Specialty |
| 207NI0002X | Clinical & Laboratory Dermatological Immunology Physician | Other Specialty |
| 207ND0900X | Dermatopathology Physician | Other Specialty |
| 207N00000X | Dermatology Physician | Other Specialty |
| 207LP3000X | Pediatric Anesthesiology Physician | Other Specialty |
| 207LP2900X | Pain Medicine (Anesthesiology) Physician | Other Specialty |
| 207LC0200X | Critical Care Medicine (Anesthesiology) Physician | Other Specialty |
| 207LA0401X | Addiction Medicine (Anesthesiology) Physician | Other Specialty |
| 207L00000X | Anesthesiology Physician | Other Specialty |
| 207KI0005X | Clinical & Laboratory Immunology (Allergy & Immunology) Physician | Other Specialty |
| 207KA0200X | Allergy Physician | Other Specialty |
| 207K00000X | Allergy & Immunology Physician | Other Specialty |
| 204R00000X | Electrodiagnostic Medicine Physician | Other Specialty |
| 204F00000X | Transplant Surgery Physician | Other Specialty |
| 204E00000X | Oral & Maxillofacial Surgery (D.M.D.) | Other Specialty |
| 204D00000X | Neuromusculoskeletal Medicine & OMM Physician | Other Specialty |
| 204C00000X | Sports Medicine (Neuromusculoskeletal Medicine) Physician | Other Specialty |
| 202K00000X | Phlebology Physician | Other Specialty |
| 202D00000X | Integrative Medicine Physician | Other Specialty |
| 202C00000X | Independent Medical Examiner Physician | Other Specialty |
| 174400000X | Specialist | Other Specialty |
| 173000000X | Legal Medicine | Other Specialty |
| 1835P2201X | Ambulatory Care Pharmacist | Pharmacist |

© 2025 Hammond G et al. *JAMA Health Forum*.

| | | |
|---|---|---|
| 1835P1300X | Psychiatric Pharmacist | Pharmacist |
| 1835P1200X | Pharmacotherapy Pharmacist | Pharmacist |
| 1835P0018X | Pharmacist Clinician (PhC)/ Clinical Pharmacy Specialist | Pharmacist |
| 183500000X | Pharmacist | Pharmacist |
| 3336S0011X | Specialty Pharmacy | Pharmacy |
| 3336L0003X | Long Term Care Pharmacy | Pharmacy |
| 3336I0012X | Institutional Pharmacy | Pharmacy |
| 3336H0001X | Home Infusion Therapy Pharmacy | Pharmacy |
| 3336C0004X | Compounding Pharmacy | Pharmacy |
| 3336C0003X | Community/Retail Pharmacy | Pharmacy |
| 3336C0002X | Clinic Pharmacy | Pharmacy |
| 333600000X | Pharmacy | Pharmacy |
| 332900000X | Non-Pharmacy Dispensing Site | Pharmacy |
| 227800000X | Certified Respiratory Therapist | Physical Therapy Occupational Therapy |
| 226300000X | Kinesiotherapist | Physical Therapy Occupational Therapy |
| 225XP0200X | Pediatric Occupational Therapist | Physical Therapy Occupational Therapy |
| 225XP0019X | Physical Rehabilitation Occupational Therapist | Physical Therapy Occupational Therapy |
| 225XN1300X | Neurorehabilitation Occupational Therapist | Physical Therapy Occupational Therapy |
| 225XL0004X | Low Vision Occupational Therapist | Physical Therapy Occupational Therapy |
| 225XH1200X | Hand Occupational Therapist | Physical Therapy Occupational Therapy |
| 225XG0600X | Gerontology Occupational Therapist | Physical Therapy Occupational Therapy |
| 225XE1200X | Ergonomics Occupational Therapist | Physical Therapy Occupational Therapy |
| 225X00000X | Occupational Therapist | Physical Therapy Occupational Therapy |
| 225700000X | Massage Therapist | Physical Therapy Occupational Therapy |
| 2251X0800X | Orthopedic Physical Therapist | Physical Therapy Occupational Therapy |
| 2251S0007X | Sports Physical Therapist | Physical Therapy Occupational |

© 2025 Hammond G et al. *JAMA Health Forum*.

| | | |
|---|---|---|
| | | Therapy |
| 2251P0200X | Pediatric Physical Therapist | Physical Therapy Occupational Therapy |
| 2251N0400X | Neurology Physical Therapist | Physical Therapy Occupational Therapy |
| 2251H1300X | Human Factors Physical Therapist | Physical Therapy Occupational Therapy |
| 2251H1200X | Hand Physical Therapist | Physical Therapy Occupational Therapy |
| 2251G0304X | Geriatric Physical Therapist | Physical Therapy Occupational Therapy |
| 2251E1300X | Clinical Electrophysiology Physical Therapist | Physical Therapy Occupational Therapy |
| 2251E1200X | Ergonomics Physical Therapist | Physical Therapy Occupational Therapy |
| 2251C2600X | Cardiopulmonary Physical Therapist | Physical Therapy Occupational Therapy |
| 225100000X | Physical Therapist | Physical Therapy Occupational Therapy |
| 172M00000X | Mechanotherapist | Physical Therapy Occupational Therapy |
| 213ES0131X | Foot Surgery Podiatrist | Podiatry |
| 213ES0103X | Foot & Ankle Surgery Podiatrist | Podiatry |
| 213ES0000X | Sports Medicine Podiatrist | Podiatry |
| 213ER0200X | Radiology Podiatrist | Podiatry |
| 213EP1101X | Primary Podiatric Medicine Podiatrist | Podiatry |
| 213EP0504X | Public Medicine Podiatrist | Podiatry |
| 213E00000X | Podiatrist | Podiatry |
| 208D00000X | General Practice Physician | Primary Care |
| 2083P0901X | Public Health & General Preventive Medicine Physician | Primary Care |
| 2083P0500X | Preventive Medicine/Occupational Environmental Medicine Physician | Primary Care |
| 2083B0002X | Obesity Medicine (Preventive Medicine) Physician | Primary Care |
| 208000000X | Pediatrics Physician | Primary Care |
| 207VX0000X | Obstetrics Physician | Primary Care |
| 207VG0400X | Gynecology Physician | Primary Care |
| 207V00000X | Obstetrics & Gynecology Physician | Primary Care |
| 207R00000X | Internal Medicine Physician | Primary Care |
| 207QB0002X | Obesity Medicine (Family Medicine) Physician | Primary Care |

© 2025 Hammond G et al. *JAMA Health Forum*.

| | | |
|---|---|---|
| 207QA0505X | Adult Medicine Physician | Primary Care |
| 207QA0000X | Adolescent Medicine (Family Medicine) Physician | Primary Care |
| 207Q00000X | Family Medicine Physician | Primary Care |
| 373H00000X | Day Training/Habilitation Specialist | Rehabilitation |
| 225C00000X | Rehabilitation Counselor | Rehabilitation |
| 225400000X | Rehabilitation Practitioner | Rehabilitation |
| 282NR1301X | Rural Acute Care Hospital | Rural Clinic |
| 261QR1300X | Rural Health Clinic/Center | Rural Clinic |
| 390200000X | Student in an Organized Health Care Education/Training Program | Student |
| 208C00000X | Colon & Rectal Surgery Physician | Surgery |
| 2086X0206X | Surgical Oncology Physician | Surgery |
| 2086S0129X | Vascular Surgery Physician | Surgery |
| 2086S0127X | Trauma Surgery Physician | Surgery |
| 2086S0122X | Plastic and Reconstructive Surgery Physician | Surgery |
| 2086S0120X | Pediatric Surgery Physician | Surgery |
| 2086S0105X | Surgery of the Hand (Surgery) Physician | Surgery |
| 2086S0102X | Surgical Critical Care Physician | Surgery |
| 2086H0002X | Hospice and Palliative Medicine (Surgery) Physician | Surgery |
| 208600000X | Surgery Physician | Surgery |
| 2082S0105X | Surgery of the Hand (Plastic Surgery) Physician | Surgery |
| 2082S0099X | Plastic Surgery Within the Head and Neck (Plastic Surgery) Physician | Surgery |
| 208200000X | Plastic Surgery Physician | Surgery |
| 207YS0123X | Facial Plastic Surgery Physician | Surgery |
| 207XX0004X | Orthopaedic Foot and Ankle Surgery Physician | Surgery |
| 207XS0117X | Orthopaedic Surgery of the Spine Physician | Surgery |
| 207XS0106X | Orthopaedic Hand Surgery Physician | Surgery |
| 207X00000X | Orthopaedic Surgery Physician | Surgery |
| 207ND0101X | MOHS-Micrographic Surgery Physician | Surgery |

© 2025 Hammond G et al. *JAMA Health Forum*.

**eTable 4.** Expanded Participant Characteristics for ACO REACH, MSSP, and All NPPES

| | ACO REACH | MSSP ACO | All NPPES |
|---|---|---|---|
| **Type of Providers** | | | |
| Non-organizational providers | 193,119 (86%) | 930,424 (100%) | 6,710,095 (89%) |
| Organizational providers | 31,291 (14%) | 1,930 (0%) | 853,459 (11%) |
| Missing Primary Taxonomy Code | 0 (0%) | 1,777 (0%) | 258,980 (3%) |
| **Total** | 224,410 (100%) | 934,131 (100%) | 7,822,534 (100%) |
| **Non-organizational providers** | | | |
| Physicians (e.g., MD, DO) | | | |
| Primary Care (i.e., IM, FM, OB/GYN | 54,629 (28%) | 228,743 (25%) | 569,804 (8%) |
| Hospitalist | 1,475 (1%) | 12,230 (1%) | 15,735 (0%) |
| Mental Health | 2,589 (1%) | 14,809 (2%) | 72,605 (1%) |
| Surgery | 6,154 (3%) | 45,716 (5%) | 112,802 (2%) |
| Geriatrics | 812 (0%) | 2,657 (0%) | 8,132 (0%) |
| Emergency | 4,567 (2%) | 34,196 (4%) | 75,797 (1%) |
| Hospice | 348 (0%) | 1,289 (0%) | 3,686 (0%) |
| Student | 7,487 (4%) | 34,900 (4%) | 306,471 (5%) |
| Other | 34,362 (18%) | 219,547 (24%) | 632,472 (9%) |
| Advanced Practice Provider (e.g., PA, NP, CNS) | 56,238 (29%) | 251,798 (27%) | 616,730 (9%) |
| Community Health Worker | 6 (0%) | 24 (0%) | 33,032 (0%) |
| Physical Therapy/Occupation Therapy | 8,153 (4%) | 11,736 (1%) | 484,715 (7%) |
| Case Manager | 21 (0%) | 83 (0%) | 132,971 (2%) |
| Optometry | 1,201 (1%) | 2,764 (0%) | 93,499 (1%) |
| Dietician/Nutrition | 938 (0%) | 4,467 (0%) | 67,761 (1%) |
| Dental | 141 (0%) | 1,659 (0%) | 351,356 (5%) |
| Chiropractor | 145 (0%) | 845 (0%) | 156,430 (2%) |
| Nurse, Assistants, Aides, and Technicians | 7,002 (4%) | 42,952 (5%) | 897,354 (13%) |
| Mental Health (e.g., MSW, Counselors) | 6,458 (3%) | 17,616 (2%) | 1,352,586 (20%) |
| Pharmacist | 11 (0%) | 230 (0%) | 295,035 (4%) |
| Other | 382 (0%) | 2,163 (0%) | 431,122 (6%) |
| **Total** | 193,119 (100%) | 930,424 (100%) | 6,710,095 (100%) |

© 2025 Hammond G et al. *JAMA Health Forum*.

## Medicare

### Part A — Inpatient/Hospital

- **Rural Community Hospital Demonstration - 2004**
  Focused on care for small hospitals in rural areas - 2004
- **Hospital Readmissions Reduction Program (HRRP) - 2012**
  Social risk adjustment for payment and penalties - 2019
- **Hospital Value-Based Purchasing (VBP) - 2013**
  Adjustment for dual eligibility - 2026
- **Hospital-Acquired Condition (HAC) Reduction Program - 2014**
- **Skilled Nursing Facility Value-Based Purchasing (SNF VBP) Program - 2018**

### Part B — Outpatient/Medical

- **End Stage Renal Disease Quality Incentive Program (ESRD QIP) - 2012**
  Reporting for social drivers of health - 2025
- **Merit-based Incentive Payment System (MIPS) - 2017**
  Complex patient bonus for dual eligibility - 2018
- **Medicare Diabetes Prevention Program (MDPP) - 2018**
- **ESRD Treatment Choices (ETC) - 2021**
  Benchmark adjustments and additional support for providers treating patients who are dually-eligible or receive low-income subsidy - 2021
- **Guiding an Improved Dementia Experience (GUIDE) - 2024**
  Screening for social needs; referrals to community based organizations; health equity payment adjustment - 2024

### Part C — Medicare Advantage

- **Medicare Advantage - 1999**
  Health Equity Index (HEI) measure - 2024
  Health equity analysis - 2025
  HEI Star Ratings - 2027
- **Value-Based Insurance Design Model (VBID) - 2017**
  Tailored supplemental benefits (lower prescription drug costs, grocery assistance, transportation services, etc.) - 2017, expanded 2020

### Part D — Prescription Drugs

## Medicare

### Parts A & B

- **Financial Alignment Initiative for Medicare-Medicaid Enrollees - 2013**
  For individuals dually enrolled - 2013
- **Comprehensive Care for Joint Replacement Model - 2016**
  Financial risk adjustment for dual eligibility - 2021
- **Frontier Community Health Integration Project (FCHIP) Demonstration - 2016**
  Focused on access to care for rural areas - 2016
- **Home Health Value-Based Purchasing Model (HHVBP) - 2016**
- **Bundled Payments for Care Improvement Advanced (BPCI-A) - 2018**
- **Value in Opioid Use Disorder Treatment (VIT-OUD) Demonstration - 2021**
- **Enhancing Oncology - 2023**
  Additional support; health-related social needs screening - 2023
- **States Advancing All-Payer Health Equity Approaches and Development (AHEAD) - 2023**
  Health equity plan; social risk payment adjustment; social needs screening - 2023

#### Accountable Care Organizations (ACOs)

- **Medicare Shared Savings Program (MSSP) - 2012**
  Advanced Investment Payment: additional upfront support to address social needs - 2023
- **Vermont All-Payer - 2017**
- **Maryland Total Cost of Care - 2019**
- **Pennsylvania Rural Health Model (PARHM) - 2019**
  ACOs develop in rural Pennsylvania - 2019
- **Primary Care First (PCF) - 2021**
- **Kidney Care Choices (KCC) - 2022**
- **Enhancing Oncology Model (EOS) - 2023**
  Additional financial payment for treating patients dually eligible - 2023
- **Realizing Equity, Access, and Community Health (REACH) - 2023**
  Health equity benchmark adjustment; health equity plan - 2023
- **Making Primary Care (MCP) - 2024**
  Social risk payment adjustment; screening/referral; strategic plan - 2024

© 2025 Hammond G et al. *JAMA Health Forum*.

**eFigure 1.** Operational Landscape of Medicare Value-Based Payment Models

Models without a description do not have explicit equity programs. Years of program start date and equity program start date are listed next to the respective name and description.

The Centers for Medicare and Medicaid Services began launching these value-based payment models in 2005 in an effort to increase the quality of care while decreasing healthcare costs. Medicare consists of four components: Part A covers inpatient and hospitalization costs, Part B covers outpatient services, Part C is Medicare-approved private insurance, and Part D covers prescription drugs. Parts A and B, known as Traditional Medicare, negotiate a contract with ACOs, groups of physicians and hospitals, to compensate for the services providers provide to patients, sharing in financial savings and penalties.

Currently, nine models (HHVBP, HAC, SNF VBP, MDPP, BPCI-A, VIT-OUD, KCC, PCF, Maryland Total Cost of Care, and Vermont All-Payer) do not have any explicit commitments to serve socially disadvantaged communities.[43-52] While equity is not a primary aspect of their goals, other models do make an effort to serving these communities:

Three programs (FCHIP, PARHM, and Rural Community Hospital Demonstration) have a focus on rural patients. [53-55] The Financial Alignment Initiative for Medicare-Medicaid Enrollees coordinate care for dually eligible beneficiaries. [56] Two models (GUIDE and MCP) encourage community-based organization referrals. [57-58] Two models (VBID and IOTA) delivered a tailored approach. Seven models (HRRP, ETC, GUIDE, AHEAD, MCP, Comprehensive Care for Joint Replacement, and Medicare Advantage) provide social risk adjustments.[57-65] VBP will adjust for dual-eligible status in the future. [66] Five models (ETC, MIPS, EOS, ACO MSSP, and Enhancing Oncology) provide additional bonuses. [61, 67-70] Three models (GUIDE, AHEAD, and MCP) encourage social needs screening,[57, 62, 59] and ESRD QIP model will soon require the detailed reporting of social determinants of health. [71] Three models (AHEAD, MCP, and REACH) require a health equity plan. [62, 59, 72] In contrast to these other alternative-based payment programs, ACO REACH has an explicit goal of achieving health equity and adjusts quality benchmarks according to social risk.[72]

Inactive models of note include Comprehensive Primary Care Initiative (CPC) and CPC Plus, Oncology Care Model (ONC), Medicare Care Choices Model (MCCM), and ACO Investment Model. [73-74] Current Medicaid only innovation models include Integrated Care for Kids (InCK) and Maternal Opioid Use (MOM). [77-78] Newer innovation models that have been announced include Increasing Organ Transplant Access in 2025, Primary Care Flex in 2025, Transforming Maternal Health (TMaH) in 2025, Transforming Episode Accountability Model in 2026, Radiation Oncology (RO) Program with a start date to be determined. [63, 79-82]

© 2025 Hammond G et al. *JAMA Health Forum*.

**eFigure 2.** Histogram of Number of Unique States and Zip Codes in Each ACO Accounting for 95% of All NPIs, REACH, vs MSSP

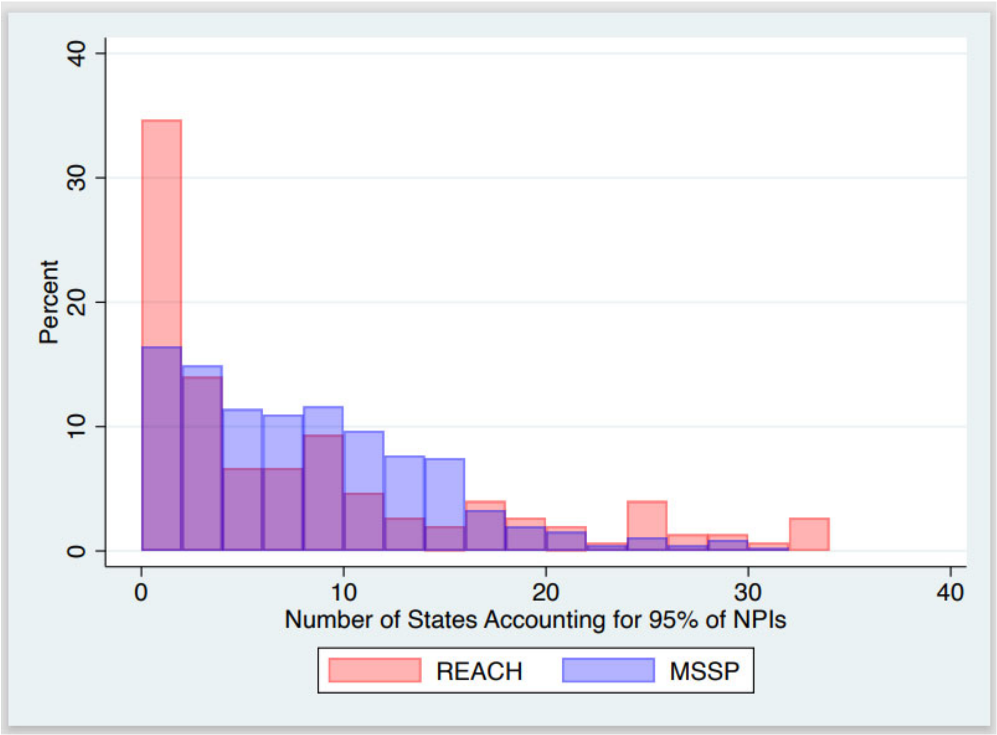


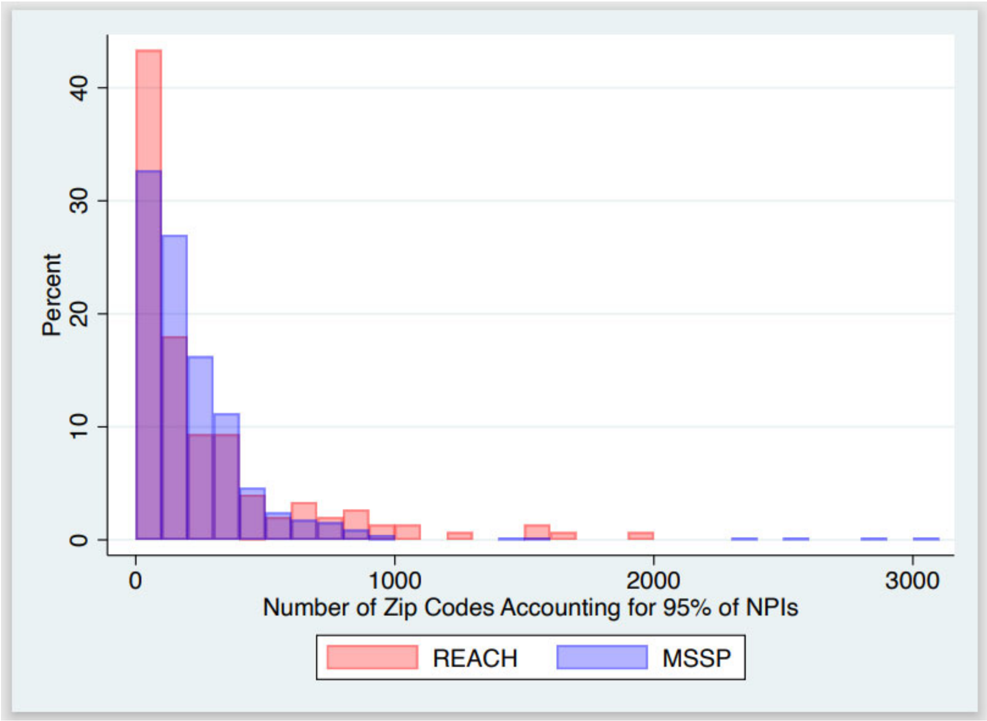


Note: Number of Unique States/Zips that account for 95% of all NPI participant in each ACO.

© 2025 Hammond G et al. *JAMA Health Forum*.

**eReferences.**


43. Expanded Home Health Value-Based Purchasing Model. Centers for Medicare and Medicaid Services. Accessed December 23, 2024. https://www.cms.gov/priorities/innovation/innovation-models/expanded-home-health-value-based-purchasing-model.
44. Hospital-Acquired Condition Reduction Program. Centers for Medicare and Medicaid Services. Accessed December 23, 2024. https://www.cms.gov/medicare/quality/value-based-programs/hospital-acquired-conditions.
45. The Skilled Nursing Facility Value-Based Purchasing (SNF VBP) Program. Centers for Medicare and Medicaid Services. Accessed December 23, 2024. https://www.cms.gov/medicare/quality/nursing-home-improvement/value-based-purchasing.
46. Medicare Diabetes Prevention Program (MDPP) Expanded Model. Centers for Medicare and Medicaid Services. Accessed December 23, 2024. https://www.cms.gov/priorities/innovation/innovation-models/medicare-diabetes-prevention-program.
47. BPCI Advanced. Centers for Medicare and Medicaid Services. Accessed December 23, 2024. https://www.cms.gov/priorities/innovation/innovation-models/bpci-advanced.
48. Value in Opioid Use Disorder Treatment (VIT-OUD) Demonstration Evaluation. Centers for Medicare and Medicaid Services. Accessed December 23, 2024. https://www.cms.gov/priorities/innovation/data-and-reports/2024/vit-intermediate-rtc.
49. Kidney Care Choices (KCC) Model. Centers for Medicare and Medicaid Services. Accessed December 23, 2024. https://www.cms.gov/priorities/innovation/innovation-models/kidney-care-choices-kcc-model.
50. Primary Care First Model. Centers for Medicare and Medicaid Services. Accessed December 23, 2024. https://www.cms.gov/priorities/innovation/innovation-models/primary-care-first-model-options.
51. Maryland Total Cost of Care Model. Centers for Medicare and Medicaid Services. Accessed December 23, 2024. https://www.cms.gov/priorities/innovation/innovation-models/md-tccm.
52. Vermont All-Payer Model. Centers for Medicare and Medicaid Services. Accessed December 23, 2024. https://www.cms.gov/priorities/innovation/innovation-models/vermont-all-payer-aco-model.
53. Frontier Community Health Integration Project Demonstration. Centers for Medicare and Medicaid Services. Accessed December 23, 2024. https://www.cms.gov/priorities/innovation/innovation-models/frontier-community-health-integration-project-demonstration.
54. Pennsylvania Rural Health Model. Centers for Medicare and Medicaid Services. Accessed December 23, 2024. https://www.cms.gov/priorities/innovation/innovation-models/pa-rural-health-model.
55. Rural Community Hospital Demonstration. Centers for Medicare and Medicaid Services. Accessed December 23, 2024. https://www.cms.gov/priorities/innovation/innovation-models/rural-community-hospital.
56. Financial Alignment Initiative for Medicare-Medicaid Enrollees. Centers for Medicare and Medicaid Services. Accessed December 23, 2024. https://www.cms.gov/priorities/innovation/innovation-models/financial-alignment.
57. Guiding an Improved Dementia Experience (GUIDE) Model. Centers for Medicare and Medicaid Services. Accessed December 23, 2024. https://www.cms.gov/priorities/innovation/innovation-models/guide.
58. Making Care Primary (MCP) Model. Centers for Medicare and Medicaid Services. Accessed December 23, 2024. https://www.cms.gov/priorities/innovation/innovation-models/making-care-primary.
59. Hospital Readmissions Reduction Program (HRRP). Centers for Medicare and Medicaid Services. Accessed December 23, 2024. https://www.cms.gov/medicare/payment/prospective-payment-systems/acute-inpatient-pps/hospital-readmissions-reduction-program-hrrp.
60. ESRD Treatment Choices (ETC) Model. Centers for Medicare and Medicaid Services. Accessed December 23, 2024. https://www.cms.gov/priorities/innovation/innovation-models/esrd-treatment-choices-model.
61. States Advancing All-Payer Health Equity Approaches and Development (AHEAD) Model. Centers for Medicare and Medicaid Services. Accessed December 23, 2024. https://www.cms.gov/priorities/innovation/innovation-models/ahead.
62. ACO Primary Care Flex Model. Centers for Medicare and Medicaid Services. Accessed December 23, 2024. https://www.cms.gov/priorities/innovation/innovation-models/aco-primary-care-flex-model.
63. Comprehensive Care for Joint Replacement Model. Centers for Medicare and Medicaid Services. Accessed December 23, 2024. https://www.cms.gov/priorities/innovation/innovation-models/cjr.
64. CMS Strategic Plan Health Equity. Centers for Medicare and Medicaid Services. Accessed December 23, 2024. https://www.cms.gov/files/document/health-equity-fact-sheet.pdf.
65. The Hospital Value-Based Purchasing (VBP) Program. Centers for Medicare and Medicaid Services. Accessed December 23, 2024. https://www.cms.gov/medicare/quality/value-based-programs/hospital-purchasing.
66. Traditional MIPS Overview. Centers for Medicare and Medicaid Services. Accessed December 23, 2024. https://qpp.cms.gov/mips/traditional-mips.
67. Enhancing Oncology Model. Centers for Medicare and Medicaid Services. Accessed December 23, 2024.


© 2025 Hammond G et al. *JAMA Health Forum*.

https://www.cms.gov/priorities/innovation/innovation-models/enhancing-oncology-model.
68. Shared Savings Program. Centers for Medicare and Medicaid Services. Accessed December 23, 2024. https://www.cms.gov/medicare/payment/fee-for-service-providers/shared-savings-program-ssp-acos.
69. Enhancing Oncology Model. Centers for Medicare and Medicaid Services. Accessed December 23, 2024. https://www.cms.gov/priorities/innovation/innovation-models/enhancing-oncology-model.
70. ESRD Quality Incentive Program. Centers for Medicare and Medicaid Services. Accessed December 23, 2024. https://www.cms.gov/medicare/quality/end-stage-renal-disease-esrd-quality-incentive-program.
71. ACO REACH. Centers for Medicare and Medicaid Services. Accessed December 23, 2024. https://www.cms.gov/priorities/innovation/innovation-models/aco-reach.
72. Comprehensive Primary Care Initiative. Centers for Medicare and Medicaid Services. Accessed December 23, 2024. https://www.cms.gov/priorities/innovation/innovation-models/comprehensive-primary-care-initiative.
73. Oncology Care Model. Centers for Medicare and Medicaid Services. Accessed December 23, 2024. https://www.cms.gov/priorities/innovation/innovation-models/oncology-care.
74. Medicare Care Choices Model. Centers for Medicare and Medicaid Services. Accessed December 23, 2024. https://www.cms.gov/priorities/innovation/innovation-models/medicare-care-choices.
75. ACO Investment Model. Centers for Medicare and Medicaid Services. Accessed December 23, 2024. https://www.cms.gov/priorities/innovation/innovation-models/aco-investment-model.
76. Integrated Care for Kids (InCK) Model. Centers for Medicare and Medicaid Services. Accessed December 23, 2024. https://www.cms.gov/priorities/innovation/innovation-models/integrated-care-for-kids-model.
77. Maternal Opioid Misuse (MOM) Model. Centers for Medicare and Medicaid Services. Accessed December 23, 2024. https://www.cms.gov/priorities/innovation/innovation-models/maternal-opioid-misuse-model.
78. Increasing Organ Transplant Access (IOTA) Model. Centers for Medicare and Medicaid Services. Accessed December 23, 2024. https://www.cms.gov/priorities/innovation/innovation-models/iota.
79. Transforming Maternal Health (TMaH) Model. Centers for Medicare and Medicaid Services. Accessed December 23, 2024. https://www.cms.gov/priorities/innovation/innovation-models/transforming-maternal-health-tmah-model.
80. Transforming Episode Accountability Model (TEAM). Centers for Medicare and Medicaid Services. Accessed December 23, 2024. https://www.cms.gov/priorities/innovation/innovation-models/team-model.
81. Radiation Oncology Model. Centers for Medicare and Medicaid Services. Accessed December 23, 2024. https://www.cms.gov/priorities/innovation/innovation-models/radiation-oncology-model.

© 2025 Hammond G et al. *JAMA Health Forum*.
