# Supplementary material for: Year 1 of Medicare’s Accountable Care Organization Realizing Equity, Access, and Community Health Model
Source: JAMA Health Forum. 2025 Apr 25;6(4):e250724. doi: 10.1001/jamahealthforum.2025.0724 (PMC12032566; doi:10.1001/jamahealthforum.2025.0724)
Supplement: Supplement 2. — Data Sharing Statement [file jamahealthforum-e250724-s002.pdf]

# Data Sharing Statement

Hammond. Year 1 of Medicare's Accountable Care Organization Realizing Equity, Access, and Community Health Model. *JAMA Health Forum*. Published April 25, 2025. doi:10.1001/jamahealthforum.2025.0724

**Data**
**Data available:** No
